# Fibrillarin Resists Cellular Senescence Via SIRT1‐Dependent Nicotinamide Metabolism and Its Inhibition Sensitizes Senolytic Therapy in Esophageal Squamous Cell Carcinoma

**DOI:** 10.1002/advs.76962

**Published:** 2026-08-10

**Authors:** Xing Jin, Hong Liu, Ling Gao, Lei Liu, Ji‐Bin Liu, Xiaoxia Jin, Ming‐Song Wang, Dan Liu, Li Feng, Wen‐Lian Chen

**Affiliations:** ^1^ Cancer Institute Longhua Hospital Shanghai University of Traditional Chinese Medicine Shanghai China; ^2^ Shanghai Frontiers Science Center of Disease and Syndrome Biology of Inflammatory Cancer Transformation Shanghai China; ^3^ Department of Thoracic Surgery The Affiliated Tumor Hospital of Nantong University Nantong China; ^4^ Cancer Institute Affiliated Tumor Hospital of Nantong University Nantong China; ^5^ Department of Pathology The Affiliated Tumor Hospital of Nantong University Nantong China; ^6^ Department of Thoracic Surgery Shanghai Ninth People's Hospital Shanghai Jiao Tong University School of Medicine Shanghai China; ^7^ Department of Medical Oncology Longhua Hospital Shanghai University of Traditional Chinese Medicine Shanghai China; ^8^ National Cancer Center/National Clinical Research Center for Cancer/Cancer Hospital Chinese Academy for Medical Sciences and Peking Union Medical College Beijing China

**Keywords:** fibrillarin, nicotinamide, senescence, SIRT1

## Abstract

The importance of fibrillarin (FBL), an established nucleolar methyltransferase, was identified in neoplastic growth of esophageal squamous cell carcinoma (ESCC), one of the most lethal malignancies worldwide. However, the underlying mechanism remains unclear. Here, by enrolling independent ESCC cohorts, we verified the negative correlation between intratumoral FBL expression and patient prognosis. Notably, comprehensive transcriptomic and metabolomic analyses indicated that *FBL* deletion elicited cell cycle arrest and cellular senescence, as well as block of nicotinamide metabolism and increase of oxidative stress. Mechanistically, FBL could bind to and stabilize SIRT1 by obstructing the ubiquitin‐proteasome‐mediated protein degradation, therefore provoking nicotinamide metabolism, sustaining redox homeostasis, and counteracting senescence. Clinically, FBL‐SIRT1 axis was augmented in tumor tissues of ESCC patients, and high intratumoral SIRT1 expression predicted poor patient survival. Finally, we found that both genetic and pharmacological inhibition of FBL could sensitize senolytic therapy for ESCC. In conclusion, we first discovered FBL as an anti‐senescent protein in tumor progression. FBL resists cellular senescence by stabilizing SIRT1 and sustaining the nicotinamide metabolism, therefore providing an optional strategy for ESCC treatment by sensitizing senolytic therapy with FBL inhibitors.

## Graphical abstract

FBL directly binds to and stabilizes SIRT1 by blocking its ubiquitin‐proteasome degradation, thereby sustaining nicotinamide metabolism and redox homeostasis to counteract cellular senescence in ESCC. Genetic and pharmacological suppression of FBL sensitizes tumor cells to senolytic therapy. This study unveils a novel anti‐senescence mechanism and provides a rationale for combining FBL‐targeted agents with senolytics as a promising therapeutic strategy against ESCC.

## 1 Introduction

Esophageal cancer is one of the most lethal carcinomas in digestive system. Pathologically, esophageal cancer is mainly classified into esophageal adenocarcinoma and esophageal squamous cell carcinoma (ESCC). Notably, ESCC accounts for approximately 90% of newly diagnosed cases, particularly in Asian populations [1, 2]. Currently, surgical resection, radiotherapy, and chemotherapy are the primary clinical treatment modalities for ESCC; however, clinical outcomes of ESCC are still unsatisfactory due to the lack of effective targeted treatments and the severe adverse effects of conventional therapies [3, 4]. Consequently, ESCC is characterized by a poor prognosis, with a median overall survival (OS) ranging from 7 to 14 months and a 5‐year OS rate below 30% [5, 6].

Abundant studies have reported that metabolic profiles of tumor cells are generally altered, and such metabolic variations are considered a core hallmark of cancer across different stages, including tumorigenesis and tumor progression [7, 8, 9]. Based on our preliminary findings, metabolite alterations may not only modify the tumor microenvironment but also influence epigenetic regulation. For instance, in our recent study, ESCC cells were found to exhibit a notably increased demand for methionine, an essential amino acid. This increase is crucial for supporting rapid cancer cell proliferation and for generating S‐Adenosyl‐Methionine (SAM), a universal methyl donor for DNA, RNA and protein methylation [10]. Other significantly activated metabolic pathways, such as proline/fatty acid/polyamine biosynthesis and glutamine/histidine/uridine metabolism, have already been reported in former researches [11, 12, 13, 14]. It is worth noting that essential metabolic enzymes may become important therapeutic targets in cancer [15, 16]. However, due to the elusive nature of key metabolic signatures in clinical ESCC tissues, no metabolism‐targeted inhibitors have yet been approved for ESCC treatment.

In our previous multi‐omics studies, we identified significant metabolic reprogramming and characterized fibrillarin (FBL) as a novel biomarker in ESCC [17]. FBL is an essential nucleolar protein that catalyzes the 2'‐O‐methylation of ribosomal RNAs. Accumulating evidence reveals that FBL could influence various processes, such as cell proliferation and drug resistance [18, 19, 20]. However, almost all studies have primarily focused on its canonical role in methylation, and the precise mechanisms by which FBL contributes to ESCC progression remain largely unclear. In this study, we further constructed two independent ESCC patient cohorts to identify and validate downstream targets of FBL.

## 2 Results

### 2.1 FBL is Highly Expressed in Tumor Tissues and Predicts Poor Prognosis in ESCC

In our previous multi‐omics research of matched tumor tissues and normal adjacent tissues (NATs) derived from 24 ESCC patients, we first unveiled that FBL is a new biomarker for ESCC. Our transcriptomics and proteomics data of these paired tissues (discovery cohort) [17] demonstrated that both mRNA and protein levels of FBL were significantly elevated in ESCC tumor tissues compared with NATs (Figure 1A,B). Additionally, similar results were observed in a public ESCC dataset GSE23400 from the GEO database (Figure 1C) and in an independent published ESCC dataset (validation cohort) by another research team [21] (Figure S1A). Furthermore, analysis of GEPIA, a database which contains datasets from TCGA and GTEx, also demonstrated up‐regulated *FBL* levels in esophageal cancer (Figure 1D). To further verify the aberrant expression of FBL in ESCC tissues, we enrolled two additional clinical ESCC cohorts (cohort 1 and cohort 2) (Tables S1 and S2). First, immunoblotting was implemented by using paired frozen tissue samples from cohort 1. The results exhibited significantly augmented FBL expression in tumor tissues compared with NATs (Figure 1E,F). Then, immunohistochemistry (IHC) assay was performed by using formalin‐fixed and paraffin‐embedded tissue specimens from cohort 1 and cohort 2. Consistent with the immunoblotting result, IHC analyses showed that FBL was highly expressed in ESCC tumor tissues (Figure 1G–J). Subsequently, we aimed to uncover the potential clinical significance of FBL in ESCC patients. As shown in Figure 1K,L, and Figure S1B, higher FBL levels predicted inferior overall survival (OS) and disease‐free survival (DFS) in patients with ESCC in both cohorts. Collectively, our results provided comprehensive evidence for the ectopic mRNA and protein expression of FBL in ESCC tumor tissues. In addition, we confirmed the reverse association between FBL levels and the prognosis of patients with ESCC.

**FIGURE 1:**
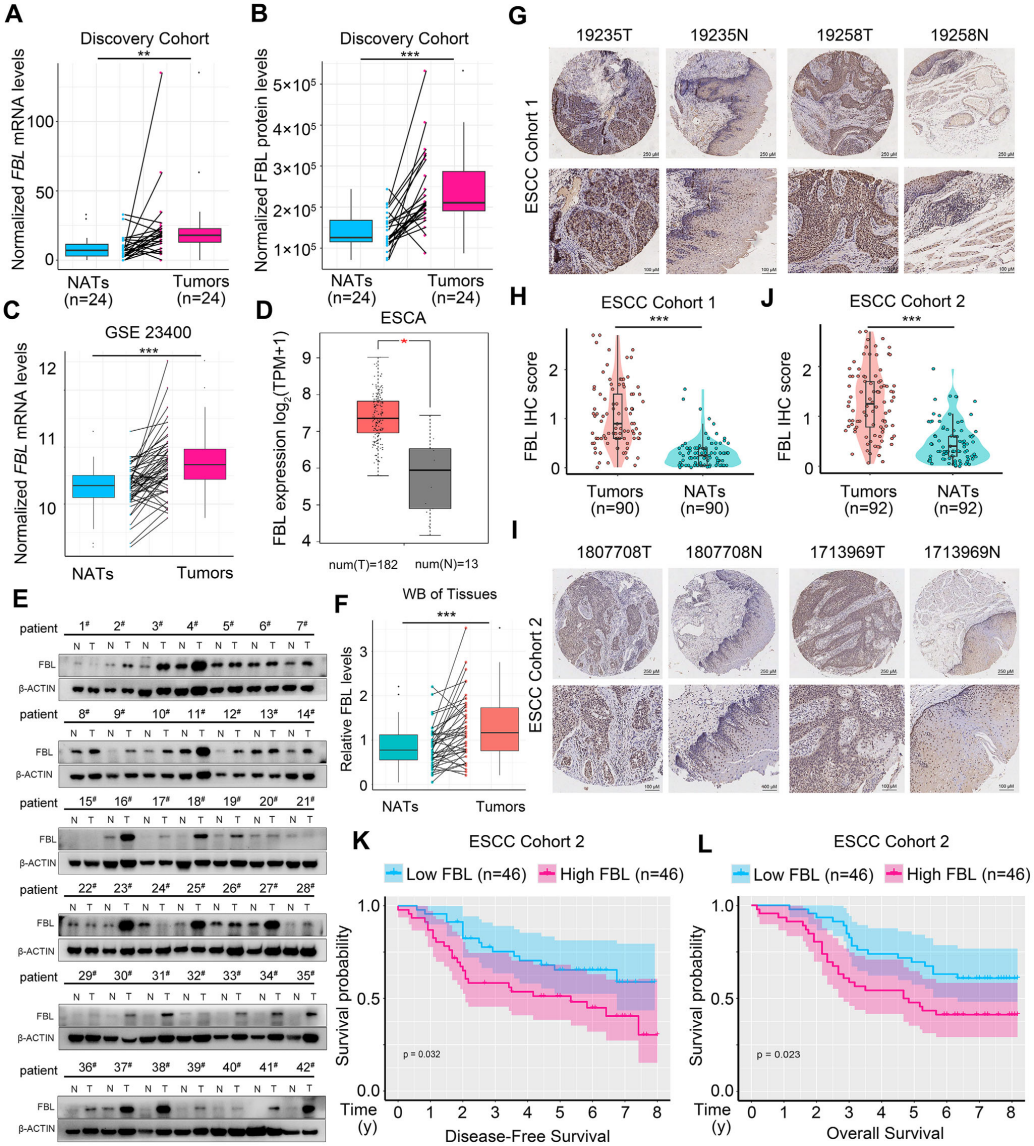
FBL is upregulated in tumor tissues and correlates with poor prognosis in ESCC. (A) The transcriptional levels of FBL between paired ESCC tumor tissues and NATs in discovery cohort (n = 24). (B) The protein levels of FBL between ESCC tumor tissues and NATs in discovery cohort. (C) The transcriptional levels of *FBL* between ESCC tumor tissues and NATs in a GEO dataset, GSE23400. (D) The transcriptional levels of *FBL* between ESCC tumor tissues and adjacent normal tissues in GEPIA dataset. (E) Western Blot revealing FBL expression between paired ESCC tissues and NATs from patient cohort 1. (F) Quantification of FBL in figure (E) by using Image J software. (G) IHC staining images showing FBL expression between paired ESCC (T) and NAT (N) tissues from patient cohort 1 (n = 90). (H) Quantification of FBL IHC staining in cohort 1. (I) IHC staining images showing FBL expression between matched ESCC (T) and NAT (N) tissues from patient cohort 2 (n = 92). (J) Quantification of FBL IHC staining in cohort 2. (K) DFS curves of ESCC patients of cohort 2 (n = 92) stratified by low and high FBL expressions from IHC staining. (L) OS curves of ESCC patients of cohort 2 (n = 92) stratified by low and high FBL expressions from IHC staining. Survival p values were computed by using log‐rank test. ^*^ *p* < 0.05, ^**^ *p* < 0.01, ^***^ *p* < 0.001, paired Wilcoxon's rank‐sum test.

Next, we interrogated whether FBL could affect the malignant phenotypes of ESCC cells. KYSE150 and Eca109, two representative ESCC cell lines, were initially enrolled for *FBL* deletion using the CRISPR‐Cas9 system (Figure S2A). We observed that *FBL* ablation obviously hindered ESCC cell proliferation and cell cycle progression (Figure S2B,C). Moreover, we utilized soft agar colony formation assay to confirm the potential oncogenic role of FBL in ESCC cells. In line with our preliminary findings, the absence of *FBL* dramatically impeded ESCC cell multiplication in soft agar (Figure S2D). Subsequently, we constructed ESCC cells with ectopic FBL expression through the CRISPR‐SAM system (Figure S2E). By contrast, FBL overexpression notably accelerated ESCC cell growth and colony formation in soft agar (Figure S2F,G). Additionally, we utilized a mouse xenograft tumor model to verify the tumor‐promoting effect of FBL. As shown in Figure S3, under *FBL* abrogation condition, the tumor growth of both KYSE150 and Eca109 cells was severely impeded. In summary, these results demonstrated that FBL expression was crucial for ESCC cell amplification both in vitro and in vivo.

### 2.2 FBL Modulates the Process of Cellular Senescence

After confirming the vital role of FBL in ESCC propagation, we sought to discover the underlying cancer‐promoting mechanism. To this end, RNA sequencing (RNA‐seq) approach was employed to measure *FBL*‐eliminated and control KYSE150 cells. Unsupervised principal component analysis (PCA) and heatmap profiling of the RNA‐seq data indicated remarkable alteration in gene expression pattern in *FBL*‐KO cells (Figure 2A,B). Subsequently, Gene Set Enrichment Analysis (GSEA) analysis revealed that cytoplasmic translation, mitochondrial translation, and ribonucleoprotein complex biogenesis were the most enriched and downregulated pathways upon *FBL* deletion (Figure 2C). To further verify these conclusions, we investigated the changes of the expression of several representative biomarkers for ribosomal and mitochondrial functions, for instance, RPS20, GUK1, and AK4. We found that all these biomarkers were prominently suppressed in *FBL*‐deficient ESCC cells, accompanied by the activation of a typical DNA damage marker, γ‐H2AX (Figure 2D). It is known that mitochondrial translation is indispensable for the synthesis of the essential oxidative phosphorylation subunits which are closely linked to the number of functioning mitochondria in cells [22]. Therefore, we observed the morphology of intracellular organelles by using an intensity diffraction tomography (IDT) system. It was found that the number of mitochondria was strikingly reduced in *FBL‐*deficient ESCC cells as relative to control cells (Figure 2E).

**FIGURE 2:**
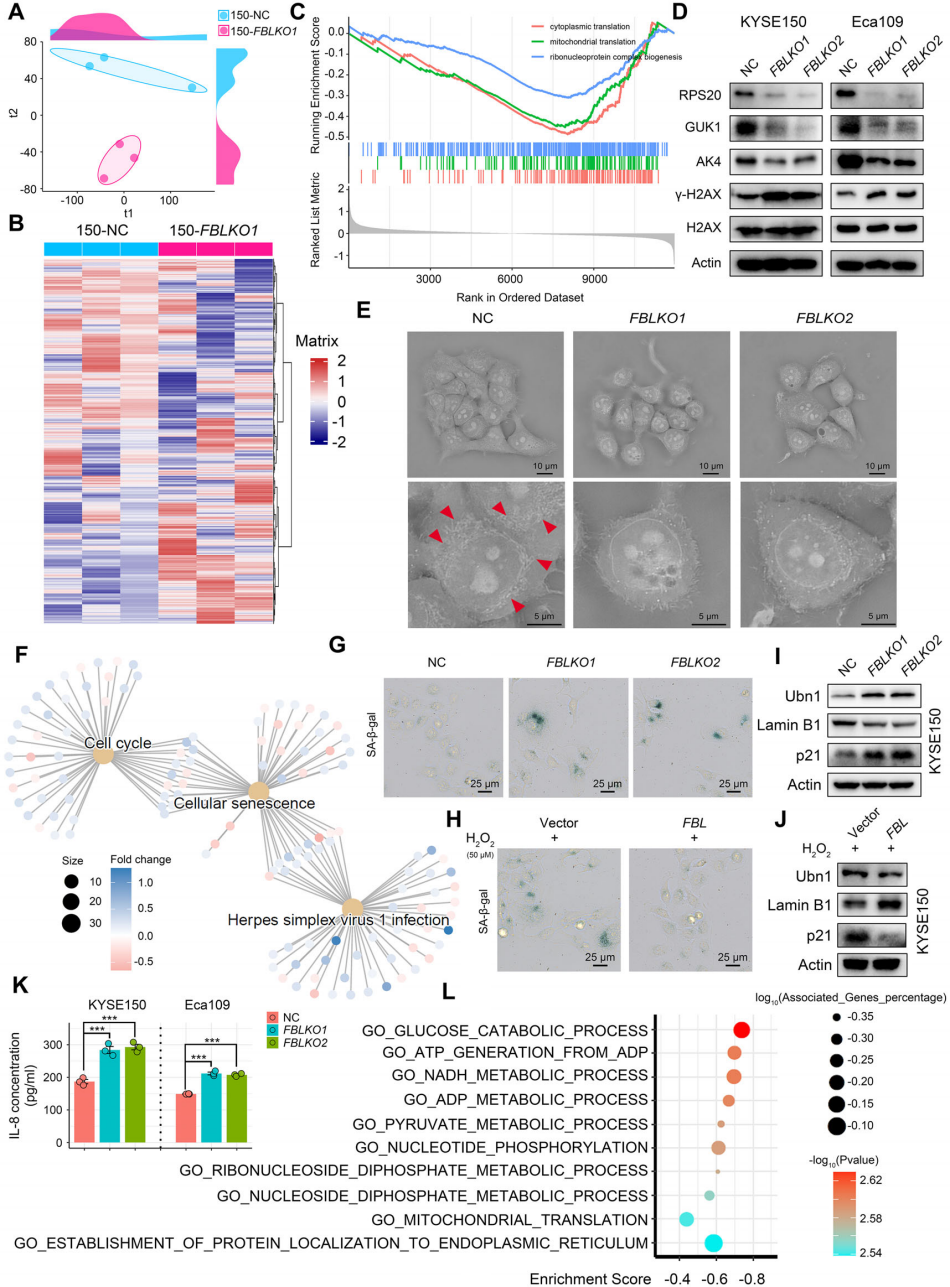
FBL modulates the process of cellular senescence. (A) PCA score plots of transcriptomics data from control and *FBL*‐deficient cells. (B) Heatmap showing all measured genes from transcriptomics analysis in control and *FBL*‐deficient KYSE150cells. (C) GSEA analysis indicating the three most significantly downregulated pathways in *FBL*‐deficient cells: cytoplasmic translation, mitochondrial translation, and ribonucleoprotein complex biogenesis. (D) Loss of *FBL* impaired the expression of markers associated with ribosome and mitochondria function, including RPS20, GUK1, and AK4. (E) Variations in mitochondrial morphology in *FBL*‐deleted ESCC cells measured by an intensity diffraction tomography system. (F) KEGG analysis revealing the three most disturbed pathways in *FBL*‐abrogated cells, including cell senescence, cell cycle, and herpes simplex virus 1 infection. (G) SA‐β‐gal staining demonstrating an increase in the proportion of SA‐β‐gal‐positive and senescent ESCC cells upon *FBL* ablation. (H) SA‐β‐gal staining revealing a decrease in the proportion of SA‐β‐gal‐positive and senescent ESCC cells upon FBL overexpression under H_2_O_2_ treatment condition (50 µM, 2 h). (I‐J) Influence on of *FBL* deletion or overexpression on the expression of cellular senescence‐associated markers, including Ubn1, lamin B1, and p21. (K) Measurement of a SASP factor IL‐8 in control and *FBL*‐abrogated ESCC cells. (L) GO analysis showing the most inhibited pathways in *FBL*‐deficient cells. Error bars represent mean ± SEM. ^*^ *p* < 0.05, ^**^ *p* < 0.01, ^***^ *p* < 0.001, Student's t‐test.

To comprehensively elucidate the FBL related mechanism, Kyoto Encyclopedia of Genes and Genomes (KEGG) pathway enrichment analysis was performed. It is noteworthy that most of disturbed transcripts were enriched in pathways linked to cellular senescence, cell cycle, and herpes simplex virus 1 infection (Figure 2F). Hence, we performed SA‐β‐galactosidase (SA‐β‐gal) staining to decipher whether *FBL* ablation could influence cellular senescence in ESCC cells. Consistent with our hypothesis, *FBL* elimination notably evoked cellular senescence (Figure 2G). In contrast, ectopic FBL expression remarkably mitigated H_2_O_2_‐elicited cellular senescence, as evidenced by a reduced number of SA‐β‐gal‐positive cells (Figure 2H). To further verify the senescent phenotype caused by *FBL* deficiency, additional senescence‐associated biomarkers were measured. *FBL* depletion led to upregulation of p21, an important cell‐cycle inhibitor, and Ubn1, a biomarker related to senescence‐associated heterochromatin foci formation (SAHF), while reducing the expression of Lamin B1, an essential component for maintaining nuclear envelope integrity (Figure 2I). Conversely, an opposite expression pattern was observed in FBL‐overexpressing cells under H_2_O_2_‐induced stress (Figure 2J). Furthermore, the secretion of interleukin‐8 (IL‐8), a prominent component of the senescence‐associated secretory phenotype (SASP), was overtly elevated by *FBL* depletion (Figure 2K). Together, these findings provided compelling evidence that loss of *FBL* promoted cellular senescence in ESCC cells.

An intriguing question was whether the SASP produced by *FBL*‐deficient cells could impact the behavior of neighboring tumor cells. To address this possibility, we constructed green fluorescent protein (GFP)‐labeled non‐targeting control (NC) KYSE150 cells and tdTomato‐labelled *FBL*‐deficient KYSE150 cells. Wild‐type (WT) KYSE150 cells were then co‐cultured with either GFP‐labeled NC cells or tdTomato‐labeled *FBL*‐deficient cells. Notably, the number of neighboring WT cells was increased progressively and significantly when co‐cultured with *FBL*‐deficient cells compared with NC cells throughout the culture period (Figure S4). These results indicated that the SASP generated by *FBL*‐deficient cells exerted a paracrine effect that promoted the propagation of neighboring ESCC cells.

As depicted above, GSEA analysis suggested that the absence of *FBL* led to mitochondrial dysfunction, potentially obstructing ATP–ADP conversion and consequently causing redox imbalance. Impaired ribosomal and mitochondrial functions are well‐established hallmarks of cellular senescence [23, 24]. Notably, the abnormal expression of ribosomal function‐related markers further supported our notion. Moreover, Gene Ontology (GO) pathway enrichment analysis indicated widespread impairment of metabolism‐related processes, such as glucose catabolic process, ATP generation from ADP, and NADH metabolic process (Figure 2L). Taken together, our results demonstrated that FBL perturbation remarkably disturbed the expression of genes associated with mitochondrial and ribosomal functions, thereby modulating cellular senescence in ESCC cells.

### 2.3 FBL is Crucial for Nicotinamide Metabolism in ESCC Cells

As described in Figure 2, metabolism‐related processes were widely disrupted, and cellular senescence were observably evoked in *FBL*‐deficient cells. To determine whether *FBL* ablation induced cellular senescence through metabolic remodeling, we examined metabolic alternations in control and *FBL*‐deficient KYSE150 cells using untargeted metabolomics. PCA and heatmap profiling of all measured metabolites manifested distinguished metabolic variation between two groups of KYSE150 cells (Figure 3A, Figure S5A). At the metabolite level, we found that the most downregulated metabolites caused by *FBL* deletion were purine nucleosides and pyridine derivatives (Figure S5B). Through KEGG pathway‐based differential abundance analysis, we identified 13 enriched metabolic pathways, with nine of these pathways exhibiting restrained activity in *FBL*‐deficient cells (Figure 3B). Notably, nicotinamide metabolism was the most significantly downregulated pathway, with approximately two‐thirds of the measured metabolites within this pathway showing markedly decreased levels. These findings indicated profound suppression of nicotinamide metabolism in *FBL*‐lacking cells. As previously described, nicotinamide metabolism, a critical pathway in redox biology, regulates oxidation homeostasis and energy conversion [25]. Consistently, volcano plot of our metabolomic data demonstrated that, under *FBL* abrogation condition, 1‐methylnicotinamide, the downstream metabolite of nicotinamide metabolism, was the most significantly decreased metabolite among all metabolites detected in the metabolomic profiling (Figure 3C). To further verify these results, targeted metabolomic analysis of nicotinamide pathway intermediates was implemented. As shown in Figure 3D, *FBL* depletion resulted in a remarkable reduction in intracellular nicotinamide (NAM) and NADH levels, accompanied by a significant increase in nicotinamide adenine dinucleotide (NAD) levels, further confirming the disruption of nicotinamide metabolism in *FBL*‐deficient cells.

**FIGURE 3:**
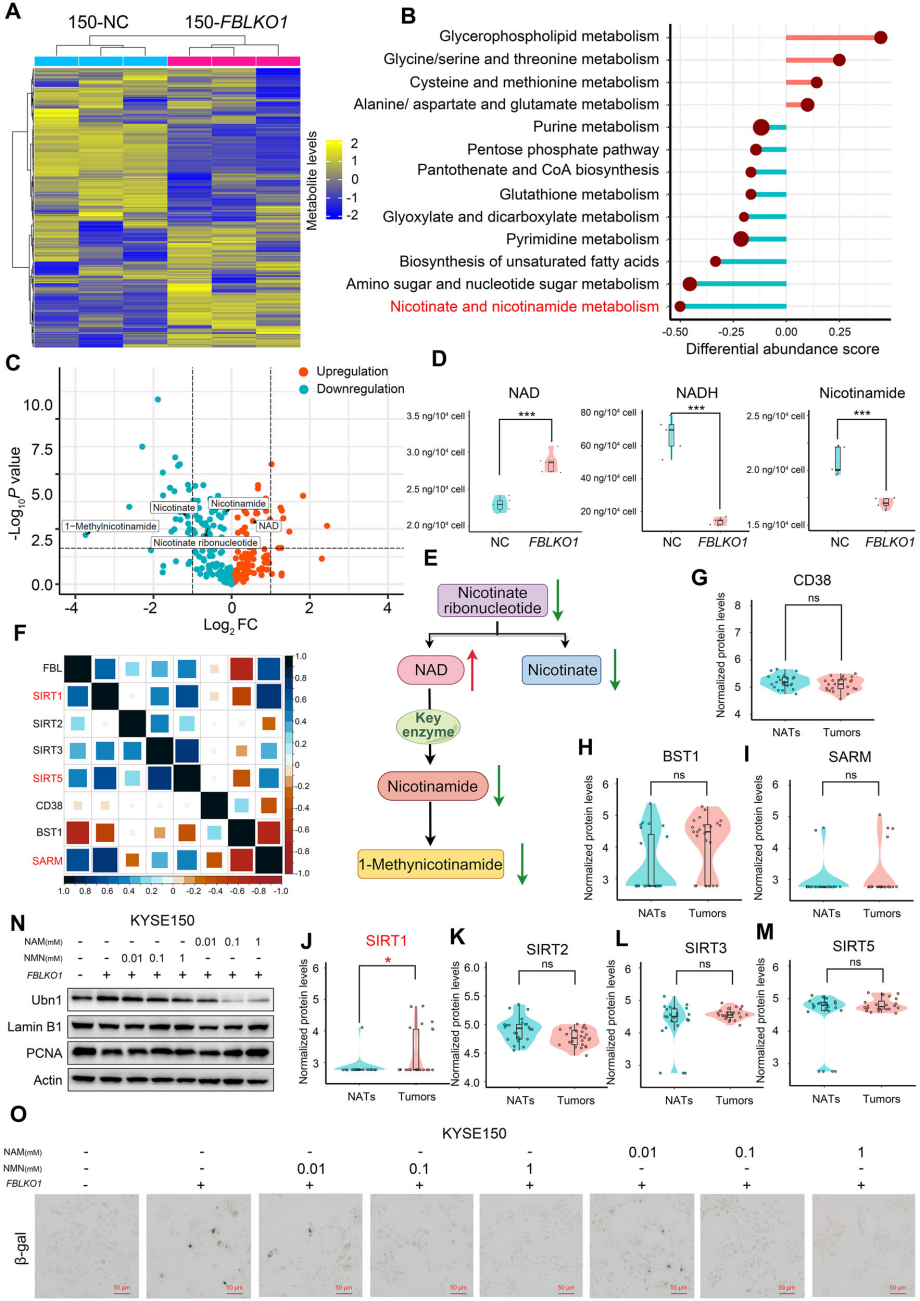
FBL is crucial for nicotinamide metabolism in ESCC cells. (A) Heatmap showing all measured metabolites between control and *FBL*‐deficient KYSE150 cells. (B) Metabolic pathway enrichment analysis of *FBL*‐deficient KYSE150 cells. The differential abundance score represents the average change of measured metabolites in a pathway in *FBL*‐deficient cells relative to control cells. Pathway size is defined by the quantity of measured metabolites participating in a particular metabolic pathway. (C) Volcano plot revealing the metabolites detected in control and *FBL*‐deficient cells. The significantly altered metabolites in nicotinamide metabolism were marked in the figure. (D) Targeted quantification of NAD, NADH, and nicotinamide in control and *FBL*‐deficient KYSE150 cells. (E) Schematic overview of nicotinamide metabolism and its associated metabolic pathway. The changes of metabolites caused by *FBL* deletion in KYSE150 cells were highlighted by arrows. (F) Correlation heatmap displaying correlation coefficients between FBL and key metabolic enzymes involved in the downstream metabolism of NAD. Proteomic data from the discovery cohort were employed for analysis. (G‐M) Expression comparison of the essential enzymes involved in the downstream metabolism of NAD between paired ESCC tissues and adjacent normal tissues. Proteomic data from the discovery cohort were used for analysis. (N) Investigation of cellular senescence‐associated with markers in *FBL*‐deficient cells following NAM or NMN supplementation. (O) SA‐β‐gal staining of *FBL*‐deficient cells revealing a decrease in the proportion of SA‐β‐gal‐positive cells after NAM or NMN addition. ^*^ *p* < 0.05, ^**^ *p* < 0.01, ^***^ *p* < 0.001; ns, no significance; Wilcoxon's rank‐sum test.

Notably, as shown in Figure 3D,E, *FBL* deletion resulted in overt reductions in nicotinate ribonucleotide, nicotinamide, nicotinate and 1‐methylnicotinamide within the nicotinamide metabolism flux. In contrast, NAD, which served both as a precursor of nicotinamide and other downstream metabolites of nicotinate ribonucleotide, was dramatically increased. These curious findings suggested that the enzymes responsible for catalyzing the conversion of NAD to nicotinamide might represent an essential regulatory node. We reasoned that *FBL* deficiency might impede this enzymatic step, leading to blockade of the conversion from NAD to NAM, which in turn caused NAM depletion and NAD accumulation.

To address above question, we compiled a list of candidate enzymes involved in nicotinamide metabolism, including CD38, BST1, SARM and members of the sirtuin family. We next performed correlation analyses using proteomic data from the discovery cohort to identify enzymes associated with FBL expression. Notably, FBL was positively correlated with SIRT1, SIRT5 and SARM, three key enzymes participated in nicotinamide metabolism (Figure 3F). Notably, among all essential enzymes involved in NAD metabolism, SIRT1 was the only enzyme that exhibited significant upregulation in clinical ESCC tissues (Figure 3G–M). Taken together, these results identified SIRT1 as a potential key downstream target through which FBL regulated nicotinamide metabolism in ESCC cells.

Finally, metabolite rescue experiments were conducted to ascertain whether nicotinamide metabolism was required for the anti‐senescent function of FBL. We found that supplementation with NAM or nicotinamide mononucleotide (NMN), two key intermediates in nicotinamide metabolism, remarkably mitigated the senescent phenotype elicited by *FBL* deficiency in ESCC cells, as evidenced by a reduction in the number of SA‐β‐gal‐positive cells and Ubn1 expression, accompanied by restoration of Lamin B1 and proliferating cell nuclear antigen (PCNA) levels (Figure 3N,O). These findings indicated that nicotinamide metabolism is essential for the anti‐senescent activity of FBL and suggested that disruption of this metabolic pathway contributes to senescence induction following *FBL* depletion.

### 2.4 FBL Enhances the Stability of SIRT1, a Key Enzyme of Nicotinamide Metabolism, Through Direct Binding

Analysis of the proteomic and transcriptomic data from the discovery and validation cohorts revealed that SIRT1 was notably upregulated in tumor tissues at the protein level but not at the mRNA level (Figure 3J, Figure S5C and Figure S6A). Therefore, we examined the correlation between FBL and SIRT1. A significant positive correlation between FBL and SIRT1 was observed at the protein level but not at the mRNA level (Figure 4A). These findings suggested that FBL might modulate SIRT1 expression in ESCC via a transcriptional‐independent mechanism. To further decipher how FBL influenced SIRT1 expression, we examined *SIRT1* mRNA levels in *FBL*‐manipulated ESCC cells and discovered no significant changes following *FBL* deletion or overexpression (Figure 4B). In contrast, *FBL* deficiency and ectopic expression resulted in marked abatement and accumulation of SIRT1 protein abundance, respectively (Figure 4C). Concordantly, SIRT1 enzymatic activity was severely impaired in *FBL*‐deficient cells (Figure 4D). In summary, these findings suggested that FBL regulated SIRT1 via a transcription‐independent mechanism. Furthermore, considering the subcellular localization patterns of FBL and SIRT1 in intracellular region (Figure 4E), we hypothesized that the two proteins might physically interacted with each other.

**FIGURE 4:**
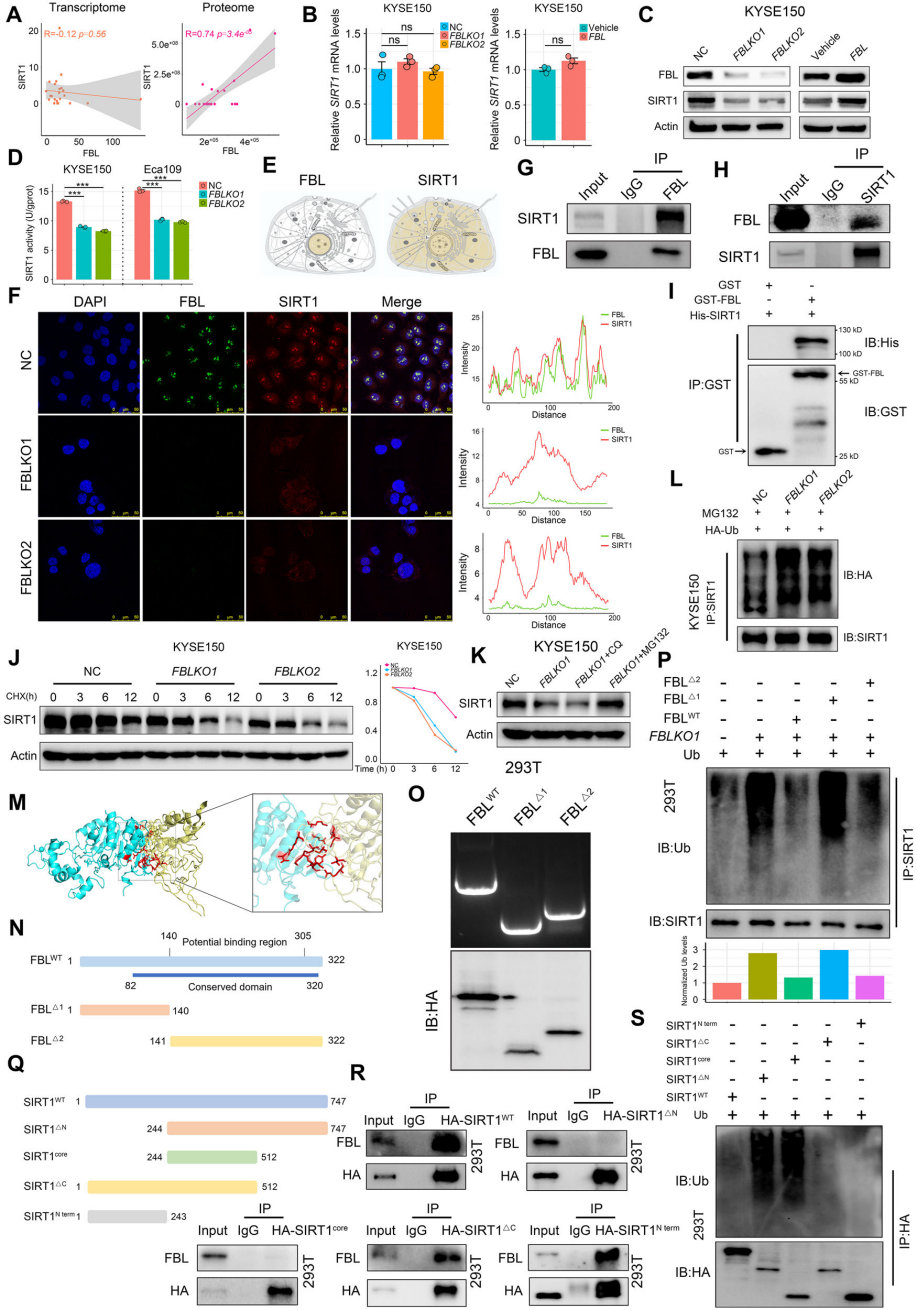
FBL enhances the stability of SIRT1 through direct binding. (A) Correlation between FBL and SIRT1 expression at both the protein and mRNA levels in ESCC tumor tissues of discovery cohort. (B) Quantitative PCR showing *SIRT1* mRNA levels in control, *FBL*‐deficient, and *FBL‐*overexpressing KYSE150 cells. (C) Western Blot revealing SIRT1 protein levels in KYSE150 cells with *FBL* knockout or ectopic expression. (D) Measurement of SIRT1 enzymatic activity in control and *FBL*‐deficient ESCC cells. (E) Subcellular localization of FBL and SIRT1 proteins in cells (Downloaded from www.uniprot.org). (F) Immunofluorescence staining showing the influence of *FBL* deletion on the nuclear co‐localization of FBL and SIRT1 in KYSE150 cells. (G‐H) Co‐immunoprecipitation assays indicating direct binding between FBL and SIRT1. (I) GST‐pull down assay demonstrating the direct interaction between FBL and SIRT1. (J) Impact of *FBL* depletion on SIRT1 protein degradation in KYSE150 cells following cycloheximide treatment (cycloheximide 50 µg/ml). (K) Rescue of *FBL* ablation‐induced SIRT1 protein degradation by the proteasome inhibitor MG132 (10 µM, 12 h), rather than the lysosome inhibitor chloroquine (10 µM, 12 h). (L) Immunoblotting assays revealing SIRT1 ubiquitination levels in control and *FBL*‐deficient cells. (M) Virtual docking analysis of the FBL–SIRT1 interaction using the HDOCK SERVER. (N) Design strategy for the construction of wildtype (WT) FBL (namely full‐length FBL) and truncated FBL mutants. (O) Expression and validation of full‐length FBL and truncated FBL mutants. (P) Influence of enforced expression of full‐length FBL and truncated FBL mutants on SIRT1 ubiquitination in *FBL*‐deficient 293T cells. (Q) Design strategy for the construction of wildtype (WT) SIRT1 (namely full‐length SIRT1) and truncated SIRT1 mutants. (R) Co‐IP assays between FBL and each SIRT1 mutant. (S) The ubiquitination abundance of enforced expressed full‐length SIRT1 and distinct truncated SIRT1 mutants in FBL‐overexpressing 293T cells. Error bars represent mean ± SEM. ^*^ *p* < 0.05, ^**^ *p* < 0.01, ^***^ *p* < 0.001; ns, no significance; Student's t‐test.

To verify this hypothesis, we subsequently investigated the co‐localization between FBL and SIRT1 in ESCC cells by immunofluorescence analysis. As shown in Figure 4F, prominent co‐localization of FBL and SIRT1 was observed in the control cells, whereas this co‐localization was diminished in *FBL*‐deficient cells. To determine whether FBL directly interacted with SIRT1, we performed co‐immunoprecipitation (Co‐IP) analysis in ESCC cells. FBL immunoprecipitation efficiently enriched SIRT1 (Figure 4G), while SIRT1 immunoprecipitation likewise pulled down FBL (Figure 4H). Moreover, a glutathione S‐transferase (GST) pull‐down assay was carried out to further validate the direct interaction between FBL and SIRT1. As revealed in Figure 4I, GST‐tagged FBL efficiently pulled down and enriched SIRT1 protein, providing additional evidence for a direct physical association between these two proteins. These results provided strong evidence that the nucleoprotein FBL could directly bind to SIRT1, a key enzyme in nicotinamide metabolism, within the nucleus of ESCC cells.

Considering that FBL directly bound to SIRT1 and exhibited a significant positive correlation with SIRT1 protein expression, we hypothesized that FBL can post‐translationally regulate SIRT1. Of note, in the presence of cycloheximide (CHX), an agent blocking the elongation phase of eukaryotic protein translation, *FBL* deletion dramatically accelerated the degradation of SIRT1, suggesting that FBL could enhance the protein stability of SIRT1 (Figure 4J, Figure S6B). Generally, protein degradation is primarily mediated by either the ubiquitin‐proteasome system or the autophagy‐lysosomal pathway. To clarify which pathway is responsible for the enhanced SIRT1 degradation caused by *FBL* deficiency, a lysosome inhibitor chloroquine (CQ) and a proteasome inhibitor MG132 were added to control and *FBL*‐depleted KYSE150 cells. As shown in Figure 4K, supplement of MG132 rather than CQ, remarkably blocked the SIRT1 degradation induced by *FBL* ablation, suggesting FBL could regulate SIRT1 via the ubiquitin‐proteasome pathway. To further verify this hypothesis, we measured the ubiquitin status of SIRT1 protein in control and *FBL*‐deficient KYSE150 cells using Co‐IP assays. The result demonstrated that *FBL* ablation dramatically intensified the ubiquitination of SIRT1 in ESCC cells (Figure 4L), indicating that FBL maintained SIRT1 protein stability by preventing ubiquitin‐dependent proteasomal degradation. An important remaining question was the identity of the E3 ubiquitin ligase responsible for SIRT1 degradation. Previous studies have identified SMURF2, Mdm2, and FZR1 as potential E3 ligases targeting SIRT1 [26, 27, 28]. To determine which ligase mediated SIRT1 degradation following *FBL* depletion, we individually silenced these candidates in *FBL*‐deficient cells. Notably, knockdown of *SMURF2*, but not *MDM*2 or *FZR*1, dramatically restored intracellular SIRT1 protein levels under condition of *FBL* loss (Figure S6C). In addition, in *FBL‐*deficient cells treated with CHX, knockdown of *SMURF2* significantly retarded SIRT1 protein degradation (Figure S6D). Collectively, these data identified SMURF2 as the principal E3 ubiquitin ligase responsible for SIRT1 degradation induced by *FBL* deficiency.

Next, we sought to discover the potential binding domain of FBL for its interaction with SIRT1. As depicted in Figure 4M, molecular docking analysis using the HDOCK system predicted the potential SIRT1‐binding region spanning the 140^th^ to 305^th^ amino acids on FBL protein. Given that full‐length FBL comprises 322 amino acids and contains a single conserved domain spanning residues 82–320, which encompasses the predicted binding region, we constructed full‐length FBL and two truncated mutants either containing or lacking this region (Figure 4N,O). Subsequently, these constructs were ectopically expressed in KYSE150 cells with *FBL* deletion, and the ubiquitination levels of SIRT1 were detected. Consistent with the docking prediction, both full‐length FBL and the truncated mutant containing the predicted binding region (FBL^△2^) could rescue the elevated ubiquitination levels of SIRT1 elicited by *FBL* loss (Figure 4P). These findings suggested that the conserved domain spanning amino acids 82–320 of FBL mediated its interaction with SIRT1 and was critical for maintaining SIRT1 protein stability.

Subsequently, we sought to determine the region of SIRT1 for its interaction with FBL. As illustrated in Figure 4Q, full‐length SIRT1 (747 amino acids) and four truncated mutants were constructed. Co‐IP assays reflected that full‐length SIRT1 (SIRT1^FL^) and two mutants, SIRT1^△C^ and SIRT1^N term^, could pull down FBL, whereas mutants of SIRT1^△N^ and SIRT1^core^ failed to enrich FBL (Figure 4R). Concordantly, under conditions of *FBL* overexpression, SIRT1^FL^, SIRT1^△C^ and SIRT1^N term^ showed minimal ubiquitination, whereas SIRT1^△N^ and SIRT1^core^ displayed substantially higher ubiquitination levels (Figure 4S). Together, these results indicated that the N‐terminal domain of SIRT1 was crucial for its binding to FBL and FBL‐mediated stabilization of the SIRT1 protein.

A previous study has established that FBL is critical for the biogenesis of ribosome, an intracellular machine responsible for translating the genetic code into proteins [29]. Therefore, we hypothesized that *FBL* deletion might also impair translational activity and thereby further reduce SIRT1 protein levels in ESCC cells. To test this hypothesis, we performed O‐Propargyl‐Puromycin (OP‐Puro) incorporation assay and noticed that *FBL* deletion resulted in an obvious global translation defect, as evidenced by the reduced insertion of OP‐Puro into nascent polypeptides (Figure S6E). This finding suggested that, in addition to facilitate SIRT1 degradation via the ubiquitin‐proteasome pathway, impaired translation caused by *FBL* loss might also contribute to the reduction of SIRT1 protein levels.

### 2.5 FBL Ablation Impairs SIRT1‐Mediated Redox Balance in ESCC Cells

Our findings revealed that FBL affects nicotinamide metabolism via regulation of SIRT1. Moreover, *SIRT1* ablation substantially hindered ESCC cell amplification (Figure 5A,B). Given the critical roles of nicotinamide metabolism and SIRT1 in redox equilibrium, we hypothesized that *FBL* abrogation might disrupt intracellular redox homeostasis. To test this hypothesis, we investigated reactive oxygen species (ROS) levels in control and *FBL*‐deficient KYSE150 cells using the DCFH‐DA probe. As shown in Figure 5C–E, both fluorescence microscopy and flow cytometry assays demonstrated that the absence of *FBL* could significantly increase the ROS levels in ESCC cells. Conversely, ectopic expression of FBL notably attenuated the abnormal elevation of ROS induced by H_2_O_2_ treatment in ESCC cells (Figure 5F–H). Together, these results indicated that FBL plays an important role in maintaining redox balance in ESCC cells.

**FIGURE 5:**
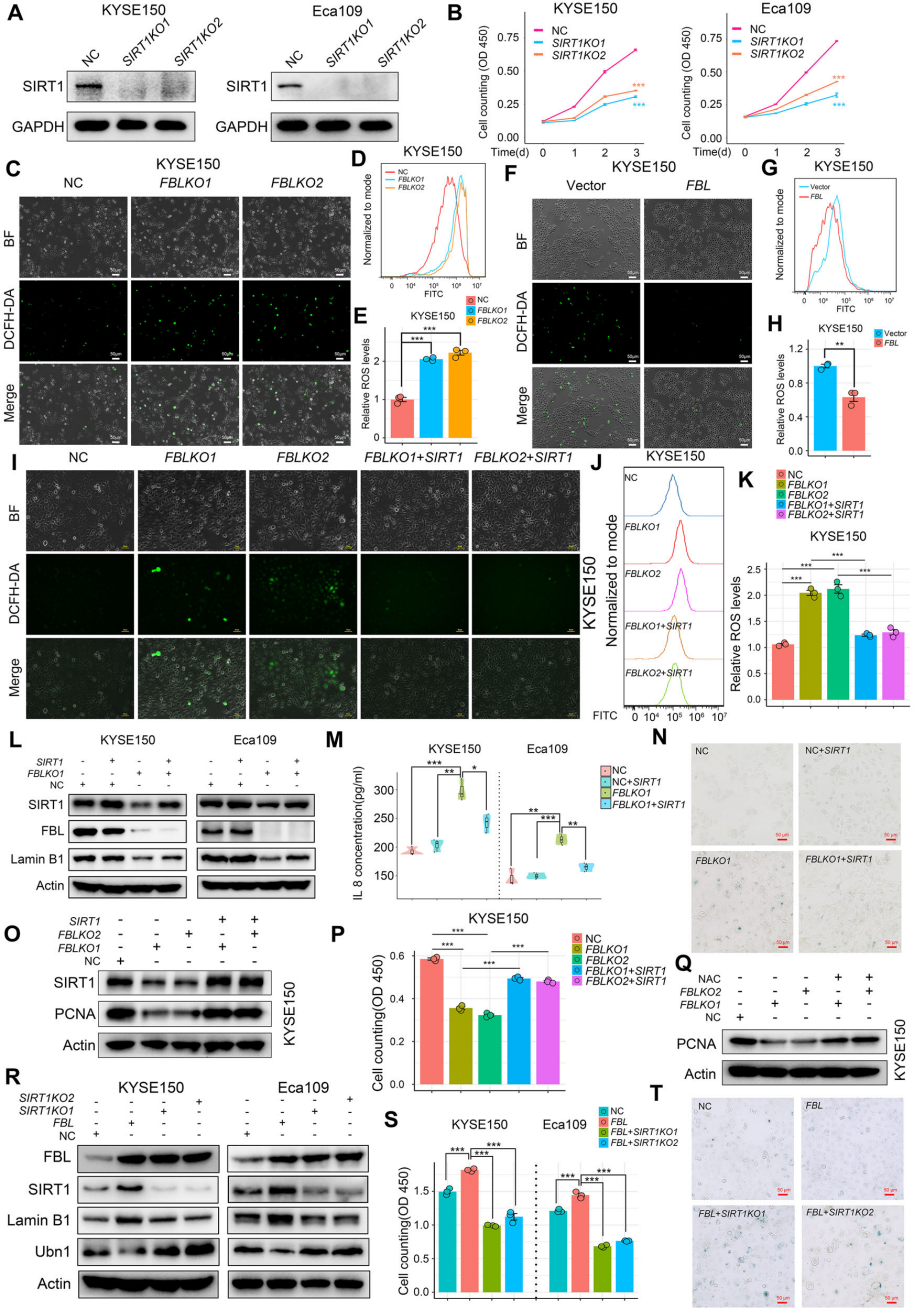
FBL ablation damages SIRT1‐mediated redox balance in ESCC cells. (A) Generation of KYSE150 and Eca109 cells with *SIRT1* deletion using the CRISPR‐Cas9 system. (B) Impact of *SIRT1* loss on ESCC cell growth. (C) Fluorescence microscopy showing increased ROS levels in *FBL*‐deficient cells compared with control cells. (D) Flow cytometry revealing altered ROS levels in control and *FBL*‐deficient ESCC cells. (E) Comparison of ROS levels between control and *FBL*‐deficient ESCC cells detected by a microplate reader. (F) Fluorescence microscopy showing ROS levels in control and FBL‐overexpressing ESCC cells with H_2_O_2_ (50 µM) treatment. (G) Flow cytometry showing altered ROS levels in control and FBL‐overexpressing ESCC cells. (H) Quantitative and comparative analyses of ROS levels between control and FBL‐overexpressing ESCC cells detected by a microplate reader. (I) Fluorescence microscopy showing ROS Levels in control cells, *FBL*‐deficient cells, and *FBL‐*deficient cells with enforced SIRT1 expression. (J) Flow cytometry exhibiting ROS levels in control cells, *FBL*‐deficient cells, and *FBL‐*deficient cells with enforced SIRT1 expression. (K) Quantitative and comparative analyses of ROS levels among control cells, *FBL*‐deficient cells, and *FBL‐*deficient cells with enforced SIRT1 expression. (L) Impact of enforced SIRT1 expression on the expression of the senescence‐related marker lamin B1 in *FBL*‐deficient ESCC cells. (M) Influence of enforced SIRT1 expression on IL‐8 secretion in *FBL*‐deficient ESCC cells. (N) SA‐β‐gal staining revealing a decrease in the number of SA‐β‐gal‐positive cells upon SIRT1 re‐expression in *FBL*‐deficient ESCC cells (O) Western blotting revealing the expression of proliferating cell nuclear antigen (PCNA) in control cells, *FBL*‐deficient cells, and *FBL‐*deficient cells with enforced SIRT1 expression. (P) Comparative analyses of cell proliferation among control cells, *FBL*‐lacking cells, and *FBL‐*lacking cells with enforced SIRT1 expression. (Q) Western blotting revealing PCNA expression in control cells, *FBL*‐lacking cells, and *FBL*‐lacking cells with NAC treatment (1 mM). (R) Changes in the expression of senescence‐associated markers upon *SIRT1* depletion in *FBL*‐overexpressing ESCC cells. (S) Variation in the proliferative ability upon *SIRT1* depletion in *FBL*‐overexpressing ESCC cells. (T) SA‐β‐gal staining demonstrating an increase in the number of SA‐β‐gal‐positive cells upon *SIRT1* ablation in *FBL*‐overexpressing cells. Error bars represent mean ± SEM. ^*^ *p* < 0.05, ^**^ *p* < 0.01, ^***^ *p* < 0.001, Student's t‐test.

Considering the interaction between FBL and SIRT1, as well as the role of FBL in retaining SIRT1 protein stability, we next investigated whether the ROS imbalance elicited by *FBL* deletion was dependent on SIRT1 degradation. Restoration of SIRT1 in *FBL*‐deficient KYSE150 cells dramatically declined the proportion of DCFH‐DA positive cells (Figure 5I). Consistently, ectopic SIRT1 expression overtly reversed the excessive ROS accumulation caused by *FBL* deficiency, as verified by flow cytometric analysis (Figure 5J,K). In summary, our findings suggested that the absence of *FBL* could obstruct nicotinamide metabolism by targeting the essential enzyme SIRT1, leading to ROS accumulation and subsequent redox disequilibrium in ESCC cells. Consistently, enforced expression of SIRT1 in *FBL*‐deficient cells dramatically relieved the senescent phenotype, as evidenced by a reduced number of SA‐β‐gal‐positive cells and decreased IL8 secretion, accompanied by restoration of Lamin B1 expression (Figure 5L–N). These results further support a critical role for the FBL–SIRT1 axis in suppressing cellular senescence in ESCC cells.

Next, we wondered whether restoration of SIRT1 expression could alleviate the impaired growth caused by *FBL* lacking. To this end, we investigated the effect of SIRT1 re‐expression on the levels of PCNA, a well‐established biomarker of cell replication, in *FBL*‐knockout (*FBL*‐KO) KYSE150 cells. As shown in Figure 5O, when SIRT1 was re‐expressed, PCNA expression was significantly restored in *FBL*‐KO KYSE150 cells. In line with this observation, proliferation assays reflected that the growth capacity of *FBL*‐KO cells was obviously rescued by SIRT1 restoration (Figure 5P). Additionally, supplement of N‐acetylcysteine (NAC), a potent antioxidant counteracting oxidative stress, similarly restore PCNA expression in *FBL*‐deficient cells (Figure 5Q). Conclusively, our results demonstrated that SIRT1 can relieve the cell growth inhibition caused by *FBL* deficiency through the restoration of redox balance.

Finally, we investigated whether *SIRT1* depletion could abolish the pro‐proliferative and anti‐senescent effects of FBL overexpression. We found that loss of *SIRT1* in FBL‐overexpressing cells markedly reduced PCNA expression and triggered a senescent phenotype, as evidenced by an increased number of SA‐β‐gal‐positive cells and elevated Ubn1 expression, accompanied by decreased Lamin B1 levels and cell counting (Figure 5R–T). In summary, these findings revealed that SIRT1 was critical for the growth‐promoting and anti‐senescent functions of FBL, further supporting a pivotal role of the FBL–SIRT1 axis in regulating ESCC cell proliferation and senescence.

### 2.6 SIRT1, A Downstream Target of FBL, Correlates With Poor Prognosis in ESCC

Our above findings identified SIRT1 as a downstream target of FBL. Hence, we hypothesized that intratumoral SIRT1 expression may be associated with clinical outcomes in patients with ESCC. To test this hypothesis, we first investigated the SIRT1 expression in ESCC cohort 1 by IHC (Figure 6A). SIRT1 levels were significantly higher in tumor tissues than in NATs (Figure 6B). Furthermore, we detected SIRT1 expression in clinical ESCC tissues and matched NATs by western blot (Figure 6C). Consistent with the IHC finding, SIRT1 expression was found to be notably increased in ESCC tumors when compared to paired NATs (Figure 6D). Subsequently, we performed correlation analysis of the same cohort to further validate the association between FBL and SIRT1 in ESCC tissues. As shown in Figure 6E, prominent positive correlation was observed between these two proteins in tumors. Then we explored the prognostic value of SIRT1 and discovered that high intratumoral SIRT1 expression predicted worse DFS in cohort 1 (Figure 6F).

**FIGURE 6:**
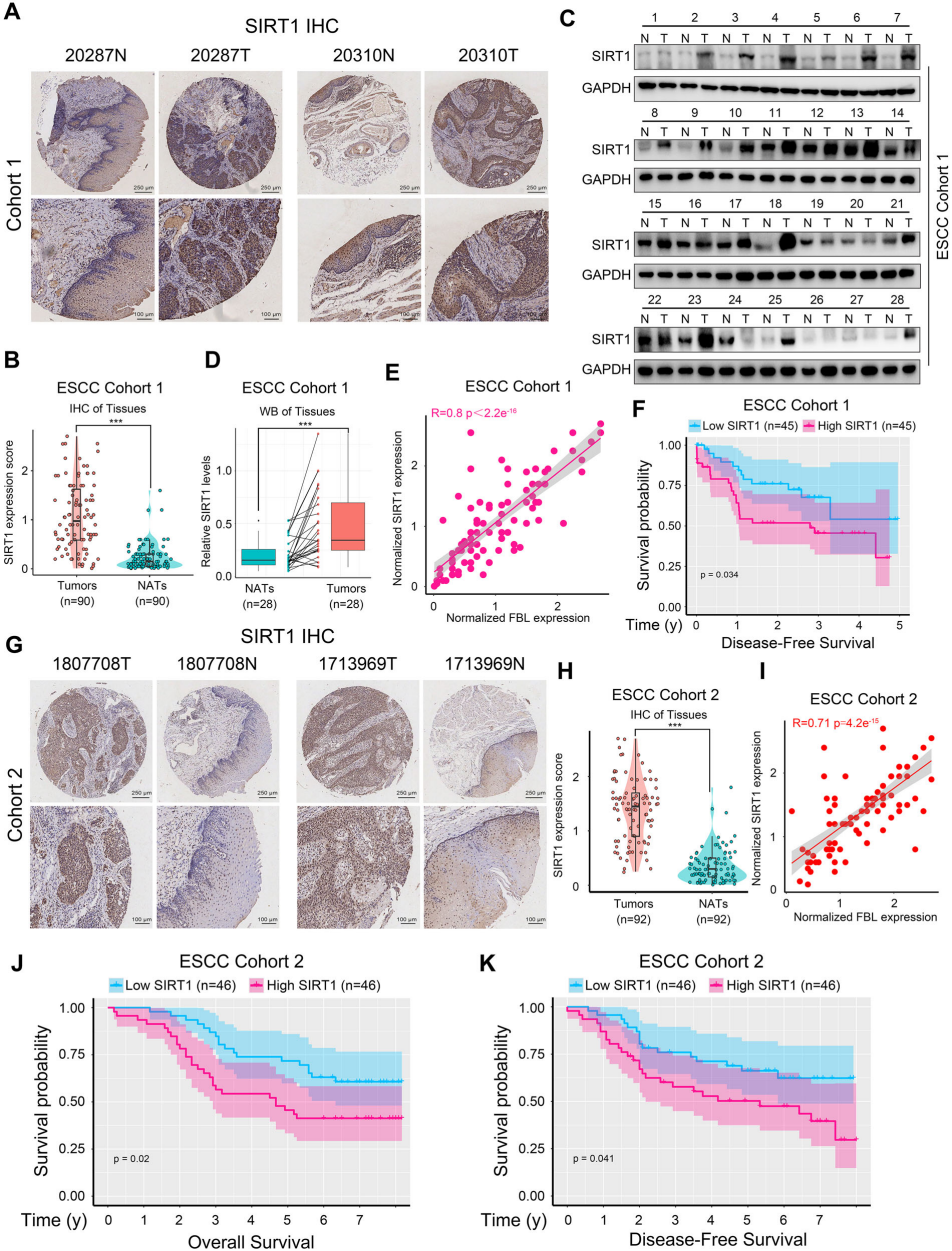
SIRT1, a downstream target of FBL, is associated with inferior prognosis in ESCC. (A) Representative IHC staining images showing SIRT1 expression between ESCC (T) and NAT (N) tissues from patient cohort 1 (n = 90). (B) Quantitative comparison of SIRT1 expression between tumor tissues and NATs in cohort 1 by IHC staining for Figure 6A. (C) Western blot revealing SIRT1 expression between ESCC tissues and NATs from ESCC cohort 1. (D) Quantitative comparison of SIRT1 expression between tumor tissues and NATs in cohort 1 for Figure 6C. (E) Correlation analysis between FBL and SIRT1 in clinical ESCC tissues derived from cohort 1. FBL and SIRT1 expression levels were quantified based on IHC staining scores. Correlation coefficient (R) values and corresponding p values were determined by using Spearman correlation analysis. Shaded area represented the 95% confidential interval of the fitted regression line. (F) DFS curves of ESCC patients stratified by low and high SIRT1 expression in cohort 1 based on IHC staining scores. (G) Representative IHC staining images showing SIRT1 expression between ESCC (T) and NAT (N) tissues from patient cohort 2 (n = 92). (H) Quantitative comparison of SIRT1 expression between tumor tissues and NATs in cohort 2 by IHC staining. (I) Correlation analysis between FBL and SIRT1 in clinical ESCC tissues derived from cohort 2. (J) OS curves of ESCC patients stratified by low and high SIRT1 expression in cohort 2 based on IHC staining scores. (K) DFS curves of ESCC patients stratified by low and high SIRT1 expression in cohort 2 based on IHC staining scores. Survival p values were computed by using log‐rank test. ^*^ *p* < 0.05, ^**^ *p* < 0.01, ^***^ *p* < 0.001, paired Wilcoxon's rank‐sum test.

Additionally, we confirmed above findings in ESCC cohort 2. In agreement with the results from cohort 1, SIRT1 expression was dramatically upregulated in ESCC tissues compared with NATs (Figure 6G,H). IHC‐based correlation analysis verified a strong positive correlation between FBL and SIRT1 expression (Figure 6I). Moreover, high intratumoral expression of SIRT1 again predicted poor OS and DFS in cohort 2 (Figure 6J,K). In summary, these results demonstrated that SIRT1, a downstream target of FBL, is highly expressed in ESCC tissues and serves as a predictor of unfavorable prognosis.

To ascertain whether SIRT1 and FBL predicted patient outcomes independently of well‐established prognostic factors, we performed multivariate Cox proportional hazards regression analysis. After adjustment for age, sex, and TNM stage, elevated intratumoral expression of both SIRT1 and FBL remained to be significantly associated with unfavorable OS (Figure S7). These findings indicated that SIRT1 and FBL are independent predictors of prognosis in ESCC patients.

### 2.7 Synergistic Anti‐Tumor Effect of FBL Targeting and Cell Senescence Scavenger in ESCC Treatment

The role of cellular senescence in cancer therapy is dual‐faceted. On the one hand, senescence exerts anti‐tumor effects by halting cell proliferation through cycle arrest; on the other hand, persistent senescent cells can promote tumor progression via senescence‐associated remodeling of the tumor microenvironment [30, 31]. Accumulated evidence reveals that targeting senescent cells by senescence scavengers represents a new and promising approach in treating various cancers [32, 33, 34]. Thus, our findings raise the possibility that targeting FBL to induce cellular senescence, in combination with senolytic therapy to eliminate senescent cells, may constitute a novel and effective therapeutic approach for ESCC.

Based on our observation that *FBL* deletion could induce cellular senescence, we next explored whether ABT‐263 [35, 36], a well‐established senescence scavenger, could synergize with *FBL* deletion in ESCC treatment. As shown in Figure 7A, absence of *FBL* induced extensive cell senescence, whereas ABT‐263 treatment could effectively eliminate senescent cells. Consistently, cell‐counting assays depicted a synergistic inhibitory effect of ABT‐263 and *FBL* deletion on ESCC cell growth, with *FBL‐*deficient cells exhibiting significantly increased sensitivity to ABT‐263 treatment (Figure 7B). Mechanistically, ABT‐263 is a potent and orally active Bcl‐2 family protein inhibitor that binds to multiple anti‐apoptotic Bcl‐2 family proteins, therefore promoting apoptosis in senescent cells [35]. To verify this mechanism, we examined the expression of apoptotic markers in ABT‐263‐treated *FBL*‐deficient ESCC cells. As shown in Figure 7C, ABT‐263 substantially increased the levels of cleaved‐PARP and cleaved‐Caspase 3 in *FBL*‐deficient cells. Moreover, flow cytometric analysis revealed a significant increase in the proportion of apoptosis cells following ABT‐263 treatment in *FBL*‐deficient KYSE150 cells, as depicted in Figure 7D,E. Collectively, these in vitro findings indicated that ABT‐263 selectively eliminates senescent cells induced by *FBL* depletion and synergistically enhances the antitumor effects of FBL targeting through the induction of apoptosis.

**FIGURE 7:**
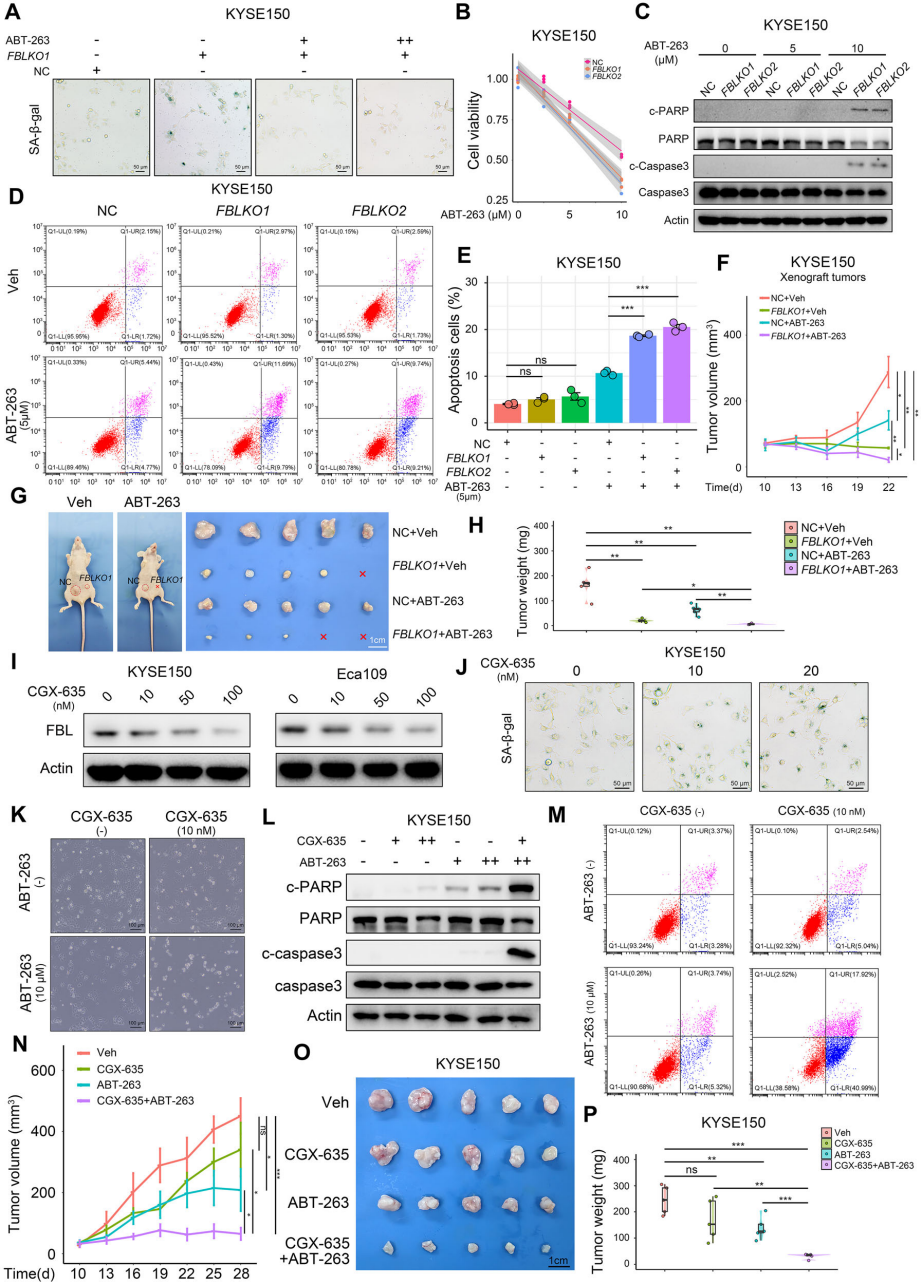
The synergistic effect of targeting FBL and cell senescence scavenger in ESCC treatment. (A) SA‐β‐galactosidase staining demonstrating the ability of the senescence scavenger ABT‐263 to eliminate senescent cells induced by *FBL* deficiency. ABT‐263: (+) 5 µM, (++) 10 µM (B) CCK8 assay comparing the sensitivity control and *FBL*‐deficient ESCC cells to ABT‐263 treatment. (C) Expression of apoptotic markers, including cleaved PARP and cleaved caspase 3, between control and *FBL*‐deficient ESCC cells with treatment of distinct concentrations of ABT‐263. (D) Flow cytometric analysis of apoptotic cells in control, *FBL*‐KO, and *FBL*‐KO ESCC cells treated with ABT‐263. (E) Quantification of apoptotic ESCC cells following *FBL* ablation alone or in combination with ABT‐263 treatment. (F‐H) Synergistic antitumor effect of *FBL* deficiency and ABT‐263 treatment on KYSE150 xenograft tumor growth. Tumor growth curves, tumor images, and statistical analysis of tumor weights were presented in F, G, and H, respectively. ABT‐263 was administered by intragastric gavage at a dose of 25 mg/kg every 3 days. (I) Influence of CGX‐635 on FBL protein levels in ESCC cells. (J) SA‐β‐galactosidase staining revealing the induction of cellular senescence by CGX‐635 treatment in KYSE150 cells. (K) Synergistic inhibitory effect of CGX‐635 (10 nM) and ABT‐263 (10 µM) on ESCC cell survival. (L) Effects of CGX‐635 and ABT‐263, alone or in combination, on apoptotic marker expression in ESCC cells. ABT‐263: (+) 5 µM, (++) 10 µM; CGX‐635: (+) 10 nM, (++) 20 nM. (M) Flow cytometric analysis showing the proportion of apoptotic ESCC cells following treatment with CGX‐635 and ABT‐263, either alone or in combination. (N‐P) Influence of CGX‐635 and ABT‐263, alone or in combination, on KYSE150 xenograft tumor growth. Tumor growth curves, tumor images, and statistical analysis of tumor weights were presented in N, O, and P, respectively. ABT‐263 was administered by intragastric gavage at a dose of 25 mg/kg every 3 days, while CGX‐635 was given by intraperitoneal injection at a dose of 0.5 mg/kg/day. Error bars represent mean ± SEM. ^*^p < 0.05, ^**^p < 0.01, ^***^p < 0.001, Student's t‐test.

Subsequently, we evaluated whether the senescence scavenger ABT‐263 could synergistically suppress tumor growth in combination with *FBL* deletion in vivo. As shown in Figure 7F–H, both *FBL* ablation and ABT‐263 treatment alone markedly inhibited tumor expansion. Importantly, the combination of *FBL* ablation and ABT‐263 produced greatest inhibitory effect on tumor growth, significantly outperforming either monotherapy. These results demonstrated a synergistic anti‐cancer effect between *FBL* targeting and senolytic therapy under in vivo conditions.

Having identified FBL as a senescence‐associated protein, we next aimed to explore the therapeutic potential of pharmacologically targeting FBL in ESCC. Previous studies have reported that CGX‐635, a selective inhibitor of FBL, exhibits the ability to directly bind to FBL and emulates the anti‐leukemia effect via *FBL* depletion [37, 38]. Concordantly, we found that CGX‐635 treatment downregulated the protein levels of FBL in a dose‐dependent manner in ESCC cells (Figure 7I). Moreover, exposure to CGX‐635 induced cellular senescence in KYSE150 cells (Figure 7J). Of note, the anti‐proliferative effect of CGX‐635 was compromised in *FBL*‐deleted cells compared with control cells (Figure S8A), suggesting that the cytotoxic activity of CGX‐635 was significantly dependent on the presence of FBL. Given its senescence‐inducing effect, we hypothesized that CGX‐635 might synergize with the senolytic agent ABT‐263 in ESCC treatment. Indeed, cell growth assays revealed a distinguished synergistic inhibitory effect of the combination treatment (Figure 7K). Furthermore, levels of cleaved‐PARP and cleaved‐Caspase 3 were dramatically raised in the combination group compared with either monotherapy (Figure 7L), suggesting enhanced induction of apoptosis. As expected, flow cytometric analysis demonstrated that the proportion of apoptotic cells in the combination group was significantly higher than in either single‐agent treatment group (Figure 7M). To quantitatively evaluate the interaction between CGX‐635 and ABT‐263, we employed the highest single agent synergy (HSA) model. The color‐gradient plots of the dose response matrix and HSA synergy scores showed a pronounced synergistic effect between these two compounds, with a mean HSA score of 8.63 (Figure S8B,C).

We next surveyed whether this synergistic effect could be recapitulated in vivo. To this end, a subcutaneous xenograft tumor model by implanting KYSE150 cells was established to evaluate the anti‐cancer efficacy of combined CGX‐635 and ABT‐263 treatment. As shown in Figure 7N–P, the combination therapy by using CGX‐635 and ABT‐263 yielded significantly greater tumor growth inhibition than either agent alone, manifesting a robust synergistic effect in vivo. Conclusively, these findings identified CGX‐635 as an attractive FBL inhibitor and suggested that its combination with the senescence scavenger ABT‐263 represented a promising therapeutic strategy for ESCC, exhibiting potent anti‐cancer activity in both in vitro and in vivo models.

Given the immunodeficient nature of the nude mouse xenograft model, we could not exclude the possibility that the therapeutic effects were partially mediated through immune‐dependent clearance of senescent ESCC cells. To address this issue, we established a subcutaneous xenograft model in immunocompetent C57BL/6 mice using AKR, a murine ESCC cell line. As shown in Figure S8D–F, combined treatment with CGX‐635 and ABT‐263 resulted in significantly greater tumor suppression than either monotherapy. Notably, the anti‐cancer efficacy of the combination therapy in immunocompetent mice was comparable to that observed in immunodeficient nude mice (Figure 7N–P, and Figure S8D–F), suggesting that ABT‐263‐mediated elimination of senescent cells occurred largely independently of host immune responses.

Lastly, we evaluated the safety profile of CGX‐635 and ABT‐263 in vivo. When compared with the control group, neither single‐agent treatment nor combination therapy caused significant changes in body weight throughout the treatment period (Figure S8G,H). In addition, histopathological examination of the liver, kidney, and spleen revealed no obvious treatment‐related abnormalities in any treatment group (Figure S8I,J). Together, these data supported the favorable tolerability and safety of CGX‐635 and ABT‐263 administration in vivo.

In conclusion, through analyses of our discovery cohort and two independent ESCC cohorts, we verified the negative correlation between intratumoral FBL expression and patient prognosis. Mechanically, FBL directly bound to and stabilized SIRT1 by obstructing its ubiquitin‐proteasome‐mediated degradation, therefore provoking nicotinamide metabolism, sustaining redox homeostasis, and counteracting cellular senescence. Furthermore, both genetic and pharmacological suppression of FBL could sensitize senolytic therapy for ESCC. To our knowledge, this study is the first to identify FBL as an anti‐senescence regulator in tumor progression. By stabilizing SIRT1 and sustaining the nicotinamide metabolism, FBL enabled ESCC cells to resist senescence. The present study not only unveiled a previously unrecognized role of FBL in ESCC pathogenesis but also provided a rationale for combining FBL‐targeted agents with senolytic therapy as an optional therapeutic strategy for FBL (Figure 8).

**FIGURE 8:**
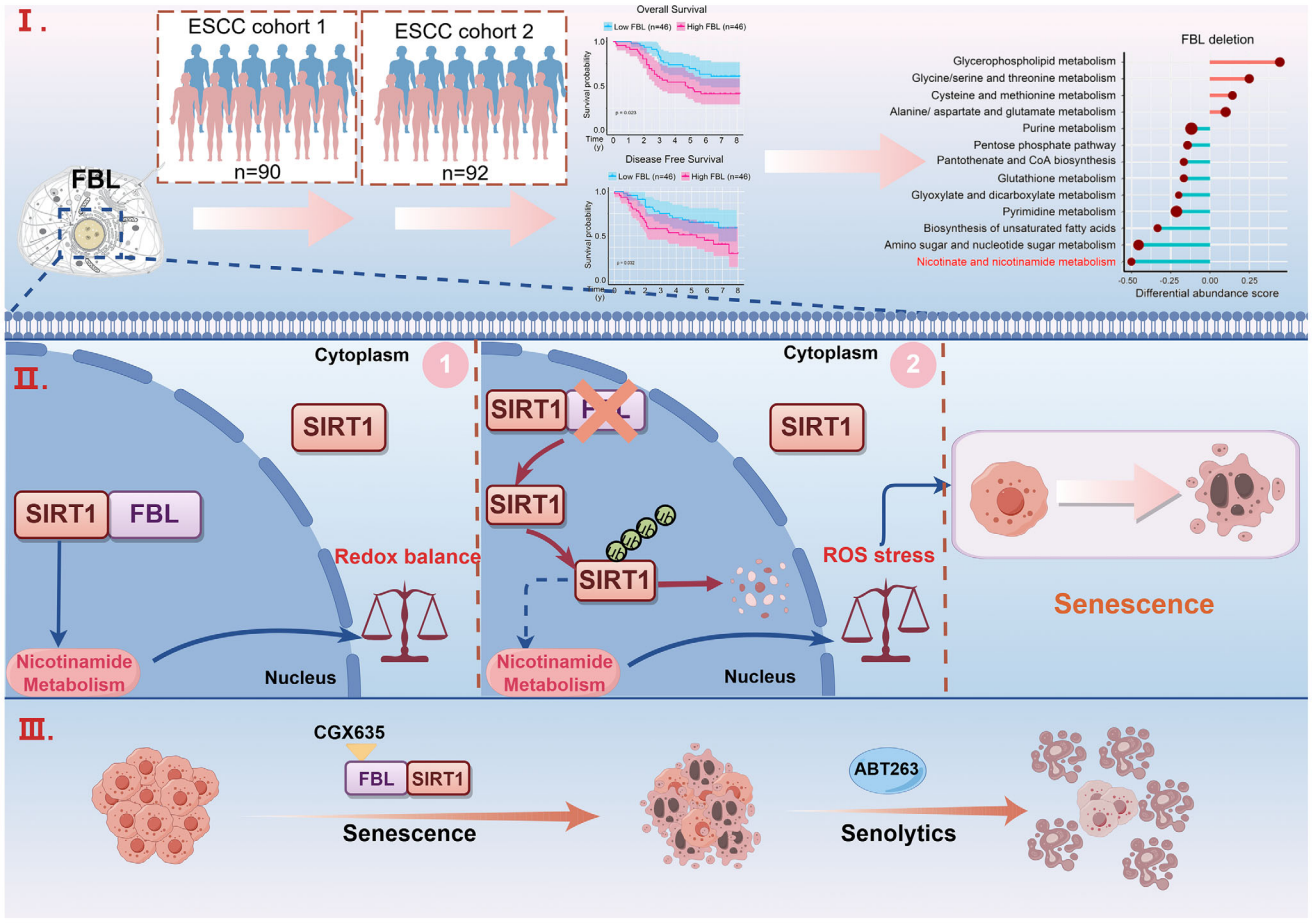
FBL resists cellular senescence and its suppression sensitizes senolytic therapy in ESCC.

## 3 Discussion

At present, the standard treatment for ESCC is multimodal therapy centered on surgical resection. It is known that squamous cell carcinoma has unique biological characteristics, while patients with advanced squamous cell carcinoma generally exhibit limited responsiveness to immunotherapy and targeted therapies, resulting in fewer treatment options than those available for patients with adenocarcinoma [39, 40]. Of note, the lack of effective therapeutic targets contributes to a poor prognosis in ESCC, with the OS rate less than 30%. In our previous study, through integrated transcriptomic, proteomic and metabolomic analyses of a discovery cohort (n = 24), we identified FBL as a novel biomarker associated with ESCC progression for the first time [17]. To further validate the clinical significance of FBL, we subsequently enrolled two independent ESCC cohorts, cohort 1 (n = 90) and cohort 2 (n = 92). Consistent with the findings from discovery cohort, both validation cohorts demonstrated remarkably upregulated FBL expression in tumor tissues compared with matched NATs. Moreover, high intratumoral expression of FBL predicted poor patient prognosis. FBL is a core component of the nucleolar small nuclear ribonucleoprotein (snRNP) particle and primarily participates in the early processing and modification of pre‐ribosomal RNA [41]. However, the mechanistic role of FBL in ESCC have not yet been elucidated. Through analyses of three independent cohorts (the discovery cohort, validation cohort 1 and validation cohort 2), we established FBL as a potential oncogene in ESCC. Functional studies further demonstrated that *FBL* depletion markedly hindered ESCC cell growth both in vitro and in vivo.

In the current study, transcriptomic analysis and experimental validation revealed that *FBL* deficiency elicits obvious cellular senescence in ESCC cells. Subsequent metabolomic analysis unveiled a new underlying mechanism by which *FBL* ablation induced cellular senescence. Notably, nicotinamide metabolism emerged as the most significantly downregulated metabolic pathway following *FBL* depletion, with SIRT1 identified as the most likely pivotal enzyme affected by this alteration. SIRT1 is a NAD‐dependent protein deacetylase that participates in the coordination of several separated cellular processes, such as energy metabolism, autophagy, and apoptosis [42, 43, 44, 45]. Our findings suggest a previously unrecognized functional link between FBL and SIRT1. As a well‐studied member of the sirtuin family, SIRT1 has been widely implicated in the regulation of senescence progression [46, 47], which is consistent with our observation that alterations in FBL expression profoundly affect senescence in ESCC.

It is worth noting that FBL regulated SIRT1 at the post‐transcriptional level rather than through transcriptional control. Furthermore, SIRT1 protein degradation was significantly accelerated in *FBL*‐deficient cells through ubiquitination‐mediated proteolysis. The underlying mechanism was elucidated, demonstrating that FBL directly binds to SIRT1 through the conserved domain encompassing amino acids 82–320 of FBL and the N‐terminal domain of SIRT1. Previous studies have illustrated that phosphorylation of SIRT1 is closely linked to its protein stability and degradation [48, 49]. In contrast, our study showed that the interaction with FBL significantly stabilized SIRT1 by obstructing ubiquitination‐mediated protein degradation. Hence, a plausible explanation is that FBL binding affects the phosphorylation status of SIRT1, thereby enhancing its stability and preventing degradation. However, this hypothesis warrants further investigation through more comprehensive mechanistic studies. In summary, our results identify FBL as a novel suppressor of cellular senescence. Loss of *FBL* promotes cellular senescence, at least in part, through destabilization of SIRT1, highlighting the FBL–SIRT1 axis as a potential regulatory pathway governing senescence in ESCC.

Considering the lack of effective therapeutic target for ESCC and the growing body of clinical evidence supporting the application of senolytic therapies [50, 51, 52], we sought to determine whether combining an FBL inhibitor with a senolytic agent could represent a new therapeutic strategy for ESCC. Recently, researchers identified the compound CGX‐635 as a novel FBL inhibitor in the study of acute myeloid leukemia [37]. This discovery prompted us to investigate whether pharmacological inhibition of FBL by CGX‐635 could induce cellular senescence in ESCC cells and, when combined with senolytic therapy, enhance antitumor efficacy. To test this hypothesis, we chose ABT‐263, a well‐established senolytic agent, and evaluate its therapeutic potency in combination with CGX‐635. Intriguingly, the combined treatment produced the greatest suppression of malignant phenotypes compared with either monotherapy alone. These findings indicate that CGX635 and ABT‐263 exert synergistic antitumor effects in ESCC, both in vitro and in vivo. Collectively, our results suggested that induction of senescence through FBL inhibition, followed by senolytic elimination of senescent tumor cells, may represent a promising therapeutic strategy for ESCC.

In conclusion, our study validated *FBL* as an oncogene whose elevated expression is associated with poor prognosis in ESCC. Mechanistically, FBL enhances SIRT1 stability through direct interaction, thereby preventing its degradation. Loss of *FBL* significantly expedites SIRT1 degradation, results in redox imbalance, and ultimately provokes cellular senescence. Furthermore, the newly identified FBL inhibitor CGX‐635 demonstrates considerable therapeutic potential in ESCC, particularly when combined with senolytics. Taken together, these findings highlight the critical role of the FBL–SIRT1 axis in regulating cellular senescence and ESCC progression. They also provide a strong rationale for exploring therapeutic strategies that integrate senescence‐inducing agents with senolytics in ESCC. Such approaches may offer a new treatment paradigm and expand therapeutic opportunities for patients with advanced ESCC who currently have limited treatment options.

## Author Contributions

Conceptualization: XJ, LF and W‐LC; Studies in vitro and in vivo: XJ, HL and LG; Patient enrollment, clinical samples, and clinical data: LL, J‐BL, XXJ and LF; Pathology, H&E, and IHC staining assays: XXJ and M‐SW; Metabolic study and data analysis: XJ and DL; Bioinformatics and statistical analysis: XJ, DL and W‐LC; Manuscript writing: XJ; Writing – review and editing: LF and W‐LC.

## Funding

This work was supported by the National Key R&D Program of China (2023YFC3503200, 2023YFC3503201), National Natural Science Foundation of China (32170778, 82304780, 82472847), Noncommunicable Chronic Diseases‐National Science and Technology Major Project (2024ZD0520903), Traditional Chinese Medicine Innovation Team and Talent Support Program‐National Traditional Chinese Medicine Multidisciplinary Cross‐Innovation Team Project (ZYYCXTD‐D‐202401), National Administration of Traditional Chinese Medicine (ZYYZDXK‐2023063), National Program for the Construction of Advantageous Traditional Chinese Medicine Specialty (XH40202‐20250904), Natural Science Foundation of Shanghai (25ZR1402486), Shanghai Three‐Year Action Plan for Inheritance, Innovation, and Development of Traditional Chinese Medicine (2025‐2027)—National Medical Center (TCM Category) Construction Project [ZY(2025‐2027)‐1‐1‐1], Tracking Program for Eastern Scholar at Shanghai Institutions of Higher Learning, Shanghai High‐level Talent Leadership Program of Traditional Chinese Medicine [ZY(2021‐2023)‐0403], the grant from Nantong Health Commission (MA2021024).

## Conflicts of Interest

The authors declare no conflicts of interest.

## Supporting information




**Supporting File**: advs76962‐sup‐0001‐SuppMat.docx.

## Data Availability

The data that support the findings of this study are available on request from the corresponding author. The data are not publicly available due to privacy or ethical restrictions.
