# Supplementary material for: Fibrillarin Resists Cellular Senescence Via SIRT1‐Dependent Nicotinamide Metabolism and Its Inhibition Sensitizes Senolytic Therapy in Esophageal Squamous Cell Carcinoma
Source: Adv Sci (Weinh). 2026 Aug 10;13(60):e76962. doi: 10.1002/advs.76962 (PMC13455048; doi:10.1002/advs.76962)
Supplement: Supplementary file 1 [file ADVS-13-e76962-s001.docx]

**Supplementary Materials and Methods**

**KEY RESOURCES TABLE**

| **REAGENT or RESOURCE** | **SOURCE** | **IDENTIFIER** |
| --- | --- | --- |
| Cell lines, Chemicals, Peptides, and antibodies |  |  |
| KYSE150 | Stem Cell Bank, Chinese Academy of Sciences | TCHu236 |
| Eca109 | SSRCC | JY334 |
| 293T | Stem Cell Bank, Chinese Academy of Sciences | GNHu17 |
| Trizol | Thermo Fisher Scientific | 15596018 |
| Polybrene | Beyotime | C0351 |
| Puromycin | Beyotime | ST551 |
| Blasticidin | Beyotime | ST018 |
| Ampicillin | Solarbio | A6920 |
| Lipofectamine 2000 | Thermo Fisher Scientific | 11668019 |
| RPMI 1640 medium | Gibco | 11875093 |
| Penicillin-streptomycin | Gibco | 15140122 |
| Fetal Bovine Serum | Biological Industries | 04-001-1ACS |
| Agarose | Sigma | 39346-81-1 |
| Cycloheximide (CHX) | MCE | HY-12320 |
| MG132 | MCE | HY-13259 |
| Chloroquine (CQ) | MCE | HY-17589A |
| Lenti-CRISPR-V2 | Addgene | 52961 |
| Lenti-SAM-V2 | Addgene | 75112 |
| psPAX2 packaging vector | Addgene | 12260 |
| pMD2.G packaging vector | Addgene | 12259 |
| BstBI | NEB | R0519 |
| XbaI | NEB | R0145 |
| DAPI | Thermofisher | 62249 |
| Prime STAR Max DNA Polymerase | Takara | R045 |
| 5×Loading buffer | Bio-rad | 1610747 |
| Anti-FBL antibody | Proteintech | 16021-1-AP |
| Anti-FBL antibody | Proteintech | 66985-1-Ig |
| Anti-PCNA antibody | Cell Signaling Technology | 13110 |
| Anti-SIRT1 antibody | Proteintech | 13161-1-AP |
| Anti-SIRT1 antibody | Proteintech | 60303-1-Ig |
| Anti-β-Actin antibody | Proteintech | 20536-1-AP |
| Anti-RPS20 antibody | Proteintech | 15692-1-AP |
| Anti-GUK1 antibody | Proteintech | 10755-1-AP |
| Anti-AK4 antibody | Proteintech | 13206-1-AP |
| Anti-Histone H_2_AX | Proteintech | 10856-1-AP |
| Anti-Phospho-Histone H2A.X | Proteintech | 83307-2-RR |
| Anti-PARP1 | Proteintech | 13371-1-AP |
| Anti-cleaved PARP1 | Proteintech | 60555-1-Ig |
| Anti-Caspase 3 | Proteintech | 19677-1-AP |
| Anti-cleaved Caspase 3 | Proteintech | 25128-1-AP |
| Anti-Ubiquitin antibody | Cell Signaling Technology | 3936 |
| Anti-Ubn1 antibody | Proteintech | 20363-1-AP |
| Anti-Lamin B1 antibody | Proteintech | 12987-1-AP |
| Anti-SMURF2 antibody | Proteintech | 18038-1-AP |
| Anti-MDM2 antibody | Proteintech | 27883-1-AP |
| Anti-FZR1 antibody | Proteintech | 16368-1-AP |
| Anti-GAPDH antibody | Proteintech | 60004-1-Ig |
| Anti-His antibody | HUABIO | HA601079 |
| Anti-GST antibody | HUABIO | EM80701 |
| Anti-HA antibody | HUABIO | ET1611-49 |
| Anti-mouse-IgG-HRP antibody | Cell Signaling Technology | 7076 |
| Anti-rabbit-IgG-HRP antibody | Cell Signaling Technology | 7074 |
| Anti-mouse-IgG-Cy3 | Beyotime | A0521 |
| Anti-rabbit-IgG-FITC | Beyotime | A0562 |
| Oligonucleotides (5’-3’) |  |  |
| CRISPR guide RNA for non-target control (NC):  FWD: AAGAAGAATTGGGGATGATG  REV: CATCATCCCCAATTCTTCTT | Addgene | N/A |
| CRISPR guide RNA #1 for human *FBL*:  corresponding to nt 102-121 of human *FBL* cDNA  FWD: TAAAGCCTCCGCCTCGACCT  REV: AGGTCGAGGCGGAGGCTTTA | Addgene | N/A |
| CRISPR guide RNA #2 for human *FBL*:  corresponding to nt 821-840 of human *FBL* cDNA  FWD: GGCAAACACGGCCTCGGCTG  REV: CAGCCGAGGCCGTGTTTGCC | Addgene | N/A |
| CRISPR guide RNA #1 for human *SIRT1*:  corresponding to nt 1132-1151 of human *SIRT1* cDNA  FWD: GTTGACTGTGAAGCTGTACG  REV: CGTACAGCTTCACAGTCAAC | Addgene | N/A |
| CRISPR guide RNA #2 for human *SIRT1*:  corresponding to nt 1052-1071 of human *SIRT1* cDNA  FWD: AACAGGTTGCGGGAATCCAA  REV: TTGGATTCCCGCAACCTGTT | Addgene | N/A |
| SAM guide RNA for human FBL:  targeting *FBL* promoter, NC_000019.10 chr19:39846572-39846591  FWD: CGCATTGGGGAAGAGGAAAC  REV: GTTTCCTCTTCCCCAATGCG | Addgene | N/A |
| Primers for full-length coding sequence of human *FBL*:  FWD: CGGGGTACCATGTACCCATACGACGTCCCAGACTACGCTAAGCCAGGATTCAGTCCCCG  REV: TGCTCTAGATCAGTTCTTCACCTTGGGGG | BioSune | N/A |
| Primers for sequence of human *FBL△1*:  FWD: CGGGGTACCATGTACCCATACGACGTCCCAGACTACGCTAAGCCAGGATTCAGTCCCCG  REV: TGCTCTAGATCAGAAGGGGTTCCAGGCTCGGTACTCAA | BioSune | N/A |
| Primers for sequence of human *FBL△2*:  FWD: CGGGGTACCATGTACCCATACGACGTCCCAGACTACGCTCGCTCCAAGCTAGCAGCAGCAAT  REV: TGCTCTAGATCAGTTCTTCACCTTGGGGG | BioSune | N/A |
| Primers for full-length coding sequence of human *SIRT1*:  FWD: GACTACGCCTCCCTCGGATCCATGGCGGACGAGGCGGCCCTC  REV: TATAGAATAGGGCCCTCTAGACTATGATTTGTTTGATGGATAGTTCAT | BioSune | N/A |
| Primers for sequence of human *SIRT1*-△N  FWD: GACTACGCCTCCCTCGGATCCATGGAAGATGCTGTGAAATTA  REV: TATAGAATAGGGCCCTCTAGACTATGATTTGTTTGATGGATAGTTCATG | BioSune | N/A |
| Primers for sequence of human *SIRT1*- CORE  FWD: GACTACGCCTCCCTCGGATCCATGGAAGATGCTGTGAAATTACT  REV: CTATAGAATAGGGCCCTCTAGACTATTCAGTAATTTCTGAAAGCTTTACA | BioSune | N/A |
| Primers for sequence of human *SIRT1*- △C  FWD: GACTACGCCTCCCTCGGATCCATGGCGGACGAGGCGGCCC  REV: TATAGAATAGGGCCCTCTAGACTATTCAGTAATTTCTGAAAGCTTTACAG | BioSune | N/A |
| Primers for sequence of human *SIRT1*- N-TERM  FWD: GACTACGCCTCCCTCGGATCCATGGCGGACGAGGCGGCCCTC  REV: TATAGAATAGGGCCCTCTAGACTAAATTGTATTAATATCTTTTCTTTTTTTCCTTTTTG | BioSune | N/A |
| Primers for sequence of PEGX-4T-1-GST-FBL  FWD: GATCTGGTTCCGCGTGGATCCATGAAGCCAGGATTCAGTCCCCG  REV: GTCACGATGCGGCCGCTCGAGTCAGTTCTTCACCTTGGGGGGT | BioSune | N/A |
| Primers for sequence of PET28A-His-SIRT1  FWD: CCGCGCGGCAGCCATATGGCTAGCATGACTGGTGGACAGCAAATGGGTCGCGGATCCATGGCGGACGAGGCGGCCC  REV: AGCAGCCGGATCTCAGTGGTGGTGGTGGTGGTGCTCGAGCTATGATTTGTTTGATGGATAGTTC | BioSune | N/A |
| siRNA sequence for *MDM2*: GGAUCAGGAUUCAGUUUCATT | BioSune | N/A |
| siRNA sequence for *SMURF2*: GACCUGUAUAUUGGAAAGUTT | BioSune | N/A |
| siRNA sequence for *FZR1*: UGAGAAGUCUCCCAGUCAG | BioSune | N/A |
| Primers for qRT-PCR of *SIRT1*:  FWD: TAGCCTTGTCAGATAAGGAAGGA  REV: ACAGCTTCACAGTCAACTTTGT | BioSune | N/A |
| Primers for qRT-PCR of *ACTB*:  FWD: CATGTACGTTGCTATCCAGGC  REV: CTCCTTAATGTCACGCACGAT | BioSune | N/A |
| Critical Commercial Assays |  |  |
| BCA Assay Kit | Thermo Scientific | 23225 |
| PrimeScript RT reagent Kit | Takara | RR047 |
| FITC Annexin V Apoptosis Detection Kit | BD | 556547 |
| Cell Cycle Analysis Kit | Beyotime | C1052 |
| Cell Counting Kit-8(CCK-8) | ShareBio | SB-CCK8 |
| Ultrapure RNA Kit | CWBIO | CW0581 |
| SYBR Premix Ex Taq Kit | Takara | RR390 |
| Senescence β-Galactosidase Staining Kit | Beyotime | C0602 |
| Reactive Oxygen Species Assay Kit | Beyotime | S0034 |
| IL-8 ELISA Kit | SIJING | A21117 |
| SIRT-1 Activity Assay Kit | Elabscience, | E-BC-F056 |
| Click-iT® Plus OPP Protein Synthesis Assay Kit | Thermo Fisher Scientific | C10456 |
| GST-tag Protein Purification Kit | Beyotime | P2262 |
| His-tag Protein Purification Kit | Beyotime | P2226 |
| Mouse: BALB/c-nude | Shanghai SLAC Laboratory Animal Co,Ltd | N/A |
| Mouse: C57BL/6 | Shanghai SLAC Laboratory Animal Co,Ltd | N/A |
| Software |  |  |
| R language | Open source | https://www.r-project.org/ |
| ImageJ software | Open source | https://imagej.nih.gov/ij/ |
| CRISPR Guide RNA database | Open source | www.addgene.org/ |
| FlowJo | Open source | Flowjo 7.6.2 |
| Image Lab | Bio-Rad | Image Lab Software 5.2.1 |

**RESOURCE AVAILABILITY**

**Lead contact**

Further information and requests for resources should be directed to and will be fulﬁlled by the lead contact, Wen-Lian Chen (chenwl8412@shutcm.edu.cn).

**Materials availability**

Materials and reagents used in this study are listed in the key resources table. Plasmids generated in this study are available from the lead contact upon request.

**Data and code availability**

This paper reanalyzes the transcriptomics and proteomics data of the discovery cohort (n=24). The raw data are available at https://www.biosino.org/node with accession numbers OEP002347 (discovery cohort) and supplementary information published in PMID: 34400640 (validation cohort). This paper does not report original code.

**METHOD DETAILS**

**Acquisition of clinical specimens and clinical data**

We enrolled two ESCC cohorts into this study, including cohort 1 (n=90) and cohort 2 (n=92), from the Affiliated Tumor Hospital of Nantong University (ATHNU) (Nantong, China). All patients were newly diagnosed and received no prior treatment for their disease. Notably, all participants provided informed written consent in accordance with the regulation of the Institutional Review Board of the ATHNU (Approval number 2019-022) in agreement with the Declaration of Helsinki.

In the ATHNU, ESCC tumor tissues and paired normal adjacent tissues (NATs) were harvested from patients of two cohorts when they underwent esophagectomy. Tissue samples were either flash frozen in liquid nitrogen and then transferred to -80 ℃ freezer for long-term storage, or formalin-ﬁxed parafﬁn-embedded for hematoxylin and eosin (H&E) staining and tissue microarray preparation. The H&E slides of each patient were independently reviewed by three board-certificated pathologists to assess the pathological characteristics. Acceptable ESCC tissue segments were determined by the percent viable tumor nuclei (> 80%), total cellularity (> 50%) and necrosis (< 20%).

Clinical characteristics of each patient were carefully reviewed and recorded. Overall survival (OS) and Disease-free survival (DFS) were obtained for ESCC cohorts. OS was computed using the time from the date of surgery to the date of death or the last follow-up.

**Table S1. Basic characteristics of ESCC cohort 1**

| Characteristic |  | ESCC (n = 90) |
| --- | --- | --- |
| Age, years  Median (range)  ≥ 60 years, no. (%)  < 60 years, no. (%) |  | 67(46-80)  76 (84.4)  14 (15.6) |
| Sex, no. (%)  Male  Female |  | 60 (66.7)  30 (33.3) |
| Alcohol consumption (habitual), no. (%)  Never  Previous |  | 80 (88.9)  10 (11.1) |
| Smoking status, no. (%)  Never smoker  Ex-smoker |  | 66 (73.3)  24 (26.7) |
| Histological type, no. (%)  Squamous-cell carcinoma  Other |  | 90 (100)  0 |
| Clinical stage group, no. (%)  Ⅰ  Ⅱ  Ⅲ  Ⅳ |  | 5 (5.6)  37 (41.1)  40 (44.4)  8 (8.9) |

**Table S2. Basic characteristics of ESCC cohort 2**

| Characteristic |  | ESCC (n = 92) |
| --- | --- | --- |
| Age, years  Median (range)  ≥ 60 years, no. (%)  < 60 years, no. (%) |  | 64(42-80)  67 (72.8)  25 (27.1) |
| Sex, no. (%)  Male  Female |  | 81 (88.0)  11 (12.0) |
| Alcohol consumption (habitual), no. (%)  Never  Previous |  | 47 (51.1)  45 (48.9) |
| Smoking status, no. (%)  Never smoker  Ex-smoker |  | 44 (47.8)  48 (52.2) |
| Histological type, no. (%)  Squamous-cell carcinoma  Other |  | 92 (100)  0 |
| Clinical stage group, no. (%)  Ⅰ  Ⅱ  Ⅲ  Ⅳ |  | 13 (14.1)  39 (42.4)  37 (40.2)  3 (3.3) |

**Cell culture**

Three human ESCC cell lines (KYSE150, Eca109) were enrolled into this study. All cell lines were authenticated using short tandem repeat (STR) profiling. Of note, all cell lines (and their derivatives) were maintained in RPMI 1640 with 10% fetal bovine serum (FBS), 100 U/mLpenicillin, and 100 μg/mL streptomycin at 37 ℃ in 5% CO2.

**Mouse models**

The subcutaneous xenograft tumor model was constructed using female BALB/c-nude mice. All mice were raised in well-ventilated cages at a constant temperature and humidity (23 ℃ ± 2 ℃, 50–60%) in a specific pathogen-free (SPF) controlled environment, with a standard 12–12 h light–dark cycle, and food and water were provided *ad libitum*. The weight and signs of diseases were measured in regular intervals. Subcutaneous tumor xenograft experiments were approved by the Institutional Animal Care and Use Committee of Shanghai University of Traditional Chinese Medicine (Approval number SYXK-Hu-2020-0009).

For the establishment of subcutaneous xenograft model, KYSE150 and Eca109 cells were resuspended in PBS buffer. Subsequently, 100 μL cell suspension solution containing 5 × 10^6^ cells was subcutaneously injected into the right flank of eight-week-old female BALB/c nude mice. For the investigation of the combination between CGX-635 and ABT-263 on xenograft tumor growth, 25mg/kg ABT-263 and 0.5mg/kg CGX-635 were injected in the mice every three days through gavage and intraperitoneal administration after ESCC cell implantation, respectively. Subcutaneous tumors were measured every three days using caliper. The volumes of tumors were calculated using the formula 0.5 × length × width^2^. Mice were sacrificed three weeks after cell inoculation. Xenograft tumors were harvested for imaging and weighing. Subsequently, tumor tissues were flash frozen in liquid nitrogen and stored at -80 ℃ for further analysis.

**Cell viability and cell proliferation analysis**

ESCC cells with different FBL or SIRT1 levels were cultured in RPMI 1640 medium, with 10% FBS, 100 U/mL penicillin, and 100 μg/mL streptomycin at 37 ℃ in 5% CO_2_. Cell viability and proliferation were measured using Cell Counting Kit-8 (CCK-8) or ATPLite kit following the manufacturer’s instruction.

**Molecular docking**

The full-length structures of FBL (PDB ID: 7SE7) and SIRT1 (PDB ID: 4KXQ) were downloaded from the PDB database. Protein-protein docking simulations between FBL and SIRT1 were performed using the HDOCK server (http://hdock.phys.hust.edu.cn/). The resulting docking conformations were analyzed to evaluate different binding poses, their corresponding binding affinities, and amino acid residues within an interaction distance of 5 Å. The protein structures were preprocessed and interacting residues between the two proteins were visualized using PyMOL software (version 2.5).

**Cell cycle assay**

Cells were digested with trypsin (0.5%) and washed for three times. Subsequently, each cell sample was resuspended in 200 μL PBS buffer. Next, cells were fixed in ice-cold 70% ethanol for 24 hours. After incubating with propidium iodide (PI)/RNase staining buffer for 30 minutes at RT, cell cycle feature of each sample was measured using a flow cytometer (Beckman Coulter, DxFLEX) and analyzed using the FlowJo software.

**Cell apoptosis assay**

Cells were digested with trypsin (0.5%) and washed for three times. Subsequently, each cell sample was resuspended in 200 μL PBS buffer. After incubating with Annexin V-FITC and propidium iodide (PI) staining buffer for 30 minutes at RT via Apoptosis Detection Kit, cell apoptosis of each sample was measured using a flow cytometer (Beckman Coulter, DxFLEX) and analyzed using the FlowJo software.

**Colony formation in soft agarose**

According to the manufacturer’s instructions, 2% agarose gels were added to MicroTissues® 3D Petri Dish® micro-mold to made soft agarose modules for 3D culturing of ESCC cells. Then, 2×10^4^ cells were added to each module with 1ml RPMI-1640 medium in 24-well plate. Cells were cultured for 5 days in cell incubator and the colonies were photographed via optical microscope.

**Hematoxylin & eosin and immunohistochemistry staining**

Esophageal tissue samples which were formalin-ﬁxed parafﬁn-embedded, were used for immunohistochemistry (IHC) staining as we previously described[1-3]. IHC staining was performed using antibodies of FBL and SIRT1.

Tissue specimens from cohort 1 and cohort 2 were used to construct tissue microarray. Subsequently, tissue microarray was stained with antibodies against FBL and SIRT1. Quantitative staining scores of tissue sections were determined by the percentage of positive cells and staining intensity. The range for percentage of positive cells was from 0% to 100%. While the staining intensity was defined as 0 for negative intensity, 1 for weak intensity, 2 for moderate intensity, and 3 for strong intensity. The normalized staining score of each sample was acquired by multiplying the proportion value of positive cells and the intensity score of the tissue section.

**Western blot assay**

Supernatants of cell/tissue lysates were collected via centrifugation at 12,000 × g for 10 minutes. The concentrations of the supernatants containing total proteins were determined using a BCA Kit. Protein extracts were denatured by addition of sample loading buffer followed by boiling for 10 minutes. Next, proteins were separated by SDS-PAGE, and then transferred to a PVDF membrane. After incubation with primary antibodies overnight at 4 °C, the membranes were washed and incubated with secondary antibodies. Western blot images were captured using a ChemiDocTouch Imaging System (Bio-rad).

**Quantitative reverse transcription-PCR**

Cells were lysed in Trizol reagent and total RNAs were extracted using an Ultrapure RNA Kit according to the manufacturer’s recommendations. RNAs were reversely transcribed to complementary DNA (cDNA) using a PrimeScript RT reagent Kit. Quantitative reverse transcription-PCR (qRT-PCR) assay was performed with a SYBR Premix Ex Taq Kit on an Applied Biosystems Q5 PCR machine (Applied Biosystems, Foster City, CA, USA).

**SA-β-gal staining**

ESCC cells were fixed by paraformaldehyde at room temperature for 15 mins. After washing three times by PBS, ESCC cells were stained via senescence-associated β-galactosidase (SA-β-gal) staining solution (Beyotime, C0602) at 37℃ for 12 hours according to the manufacturer’s instructions. Then senescent cells were photographed via optical microscope.

**H_2_O_2_-induced senescence**

In order to induce senescence, ESCC cells were cultured in RPMI 1640 with 10 % fetal bovine serum (FBS) and treated with exogenous H_2_O_2_ (50 μM). After 2 h, the culture medium was changed, and cells were kept for 24 h for further studies.

**ROS detection**

According to the manufacturer’s instructions (Beyotime, S0034), the DCFH-DA probe was diluted to 10μM using RPMI-1640 medium, then cells were incubated with DCFH-DA buffer for 20 mins in cell incubator. After washing three times by PBS, ESCC cells loaded with ROS probe were detected by fluorescence microscope, flow cytometer or microplate reader.

**Immunofluorescence**

Briefly, ESCC cells were fixed by 4% paraformaldehyde for 20 minutes at room temperature and washed three times with PBS. Subsequently, cells were permeabilized with 0.2% Triton X-100 in PBS for 10 minutes. After incubating with blocking buffer to reduce nonspecific binding, the appropriate concentration of FBL and SIRT1 antibodies were added to incubate with cells overnight at 4°C in a humidified chamber. Then the fluorescent secondary antibodies (FITC-anti-Rabbit IgG, Cy3-anti-Mouse IgG) were added to label the location of FBL and SIRT1, respectively. Finally, the nuclear dye DAPI (1 µg/mL) was applied to stain the nuclei and the location of FBL and SIRT1 in stained cells were visualized using confocal microscope.

**Gene knockout by CRISPR-Cas9 approach**

ESCC cell lines were used for gene knockout studies according to our previous protocols [1, 3-5]. Briefly, the guide RNA (gRNA) of each gene was inserted into a lenti-CRISPR-V2 plasmid which was digested by the BsmBI endonuclease. The lentivirus was packed as follows: lenti-CRISPR-V2 vector containing nontarget control (NC) gRNA or gRNAs of target genes was co-transfected with psPAX2 and pMD2.G plasmids into HEK 293T cell using the Lipofectamine 2000 reagent according to the manufacturer’s recommendations. After 48 hours of cell culture, the lentivirus supernatant was collected by centrifugation at 3,000 × g and filtered using a 0.45 μm filter in order to thoroughly remove HEK 293T cells. Subsequently, ESCC cells were infected with the NC lentivirus or lentivirus expressing target gRNAs in the presence of 8 μg/mL polybrene. Infected ESCC cells were screened with puromycin, for 48 hours. Of note, the following gRNAs were used in this study.

gRNA of NC: 5’-AAGAAGAATTGGGGATGATG-3’;

gRNA#1 for *FBL* (corresponding to nt 102-121 of human *FBL* cDNA):

5’- TAAAGCCTCCGCCTCGACCT -3’;

gRNA#2 for *FBL* (corresponding to nt 821-840 of human *FBL* cDNA):

5’- GGCAAACACGGCCTCGGCTG -3’;

gRNA#1 for *SIRT1* (corresponding to nt 1132-1151 of human *SIRT1* cDNA):

5’- GTTGACTGTGAAGCTGTACG -3’;

gRNA#2 for *SIRT1* (corresponding to nt 1052-1071 of human *SIRT1* cDNA):

5’- AACAGGTTGCGGGAATCCAA -3’.

**FBL overexpression**

The full-length and truncated coding sequence of *FBL* was cloned by a pair of primers which were listed as follows:

KpnI-*FBL*-forward primer:

5’-CGGGGTACCATGTACCCATACGACGTCCCA

GACTACGCTAAGCCAGGATTCAGTCCCCG-3’;

XbaI-*FBL*-reverse primer:

5’- TGCTCTAGATCAGTTCTTCACCTTGGGGG-3’

KpnI-*FBL^△1^*-forward primer:

5’-CGGGGTACCATGTACCCATACGACGTCCCA

GACTACGCTAAGCCAGGATTCAGTCCCCG-3’;

XbaI- *FBL^△1^*-reverse primer:

5’-TGCTCTAGATCAGAAGGGGTTCCAGGCTCGGTACTCAA-3’

KpnI-*FBL^△2^*-forward primer:

5’-CGGGGTACCATGTACCCATACGACGTCCCAGA

CTACGCTCGCTCCAAGCTAGCAGCAGCAAT-3’

XbaI- *FBL^△2^*-reverse primer:

5’-TGCTCTAGATCAGTTCTTCACCTTGGGGG-3’

The cDNA from KYSE150 cells were used as the template in the presence of the PrimeSTAR® Max DNA Polymerase. The sequence was then inserted into the pcDNA3.0 vector, which was pre-treated with KpnI and XbaI. Next, the negative control vector pcDNA3.0 and the recombinant vector pcDNA3.0-*FBL/* pcDNA3.0-*FBL^△1^/* pcDNA3.0-*FBL^△2^* were transfected into ESCC cells, respectively, according to the manufacturer’s instructions. The transfection was performed using a Lipofectamine 2000 Transfection Reagent following the manufacturer’s protocol.

**OP-puro incorporation assay**

OP-puro incorporation detections were performed using Click-iT® Plus OPP Protein Synthesis Assay Kit (Thermo Fisher Scientific, cat# C10456) according to the manual. Briefly, Cells were incubated with 20 µM O‑propargyl‑puromycin in complete medium at 37 °C for 30 min. After washing with PBS, cells were fixed with 4% paraformaldehyde for 15 min, permeabilized with 0.1% Triton X‑100. Incorporated OP‑puro was detected by copper(I)‑catalyzed azide‑alkyne cycloaddition (CuAAC click chemistry) using Click‑iT® reaction buffer for 30 min at RT protected from light. The nuclear dye DAPI (1 µg/mL) was applied to stain the nuclei. Fluorescence was visualized by confocal microscopy.

**IL-8 detection**

For interleukin-8 secretion analysis, 5 × 10⁵ control and *FBL*-deficient ESCC cells were seeded in 6‑cm dishes containing 5 mL complete medium and cultured for 24 h. Conditioned medium was then collected and centrifuged at 1,500 × g for 5 min at 4 °C to remove cell debris. The clarified supernatant was collected for IL-8 detection using IL-8 ELISA kit (SIJING, cat#A21117) according to the instruction.

**SIRT1 Deacetylase Activity Assay**

SIRT1 enzymatic activity was measured using a Sirtuin 1 (SIRT1) Activity Assay Kit (Elabscience, cat# E-BC-F056,) according to the manufacturer's protocol. Briefly, 1 × 10^6^ cells were harvested and washed twice with cold PBS and lysed in ice‑cold lysis buffer. Lysates were centrifuged at 12,000 × g for 10 min at 4 °C, and the supernatants were collected. Protein concentration was determined by BCA assay. Equal amounts of protein were mixed with the FRET‑based acetylated peptide substrate and NAD⁺ solution in assay buffer, then incubated at 37 °C for 30 min. The reaction was terminated by adding developer solution, and fluorescence was measured at excitation/emission = 340/440 nm. SIRT1 activity was calculated from a standard curve and normalized to protein concentration. Assays were performed in triplicate.

**GST pull-down Assay**

GST pull-down assays were performed to assess direct protein-protein interactions between GST-tagged FBL (GST-FBL) and His-tagged SIRT1 (His-SIRT1). Escherichia coli BL21(DE3) (Beyotime, cat#D1013S) cells were transformed with pGEX-4T-1-GST-FBL and pET28a-His-SIRT1 plasmids and induced with 0.1 mM IPTG at 16 °C for 16 h, respectively. Cells were harvested and lysed by sonication in lysis buffer (20 mM Tris-HCl pH 7.5, 150 mM NaCl, 1 mM DTT, 0.5% Triton X-100) supplemented with protease inhibitors (Beyotime, cat#P2262). BeyoGold™ GST-tag Purification Resin were pre-incubated with GST-FBL lysate at 4 °C overnight under gentle rotation to immobilize GST-FBL. After three washes with lysis buffer, the beads were incubated with His-SIRT1 lysate extracted by His-labeled protein Purification kit (Beyotime, cat#P2226) at 4 °C overnight under gentle rotation. Following five washes to remove unbound proteins, bound complexes were eluted with SDS-PAGE loading buffer. Proteins were resolved by SDS-PAGE and analyzed by Western blot using anti-His antibody to detect His-SIRT1 co-precipitation. GST alone served as a negative control.

**Ubiquitination assay**

Cells were lysed in NP40 buffer and the supernatant of the immunoprecipitated lysate was acquired and incubated with anti-SIRT1 antibodies under the condition of continuous shaking at 4 ℃ overnight. Then the protein A/G magnetic beads were added to each sample and shaking at 4 ℃ for 3 hours. The co-immunoprecipitated samples were then purified with wash buffer for 3 times. After centrifuging at 4 ℃, 12000g for 10 minutes, supernatant was acquired for detecting the ubiquitination level of SIRT1 by anti-Ub antibody via WB assay.

**RNA-sequencing** **and data analysis**

RNA-sequencing (RNA-seq) was performed by Cloud-Seq Biotech Inc. (Shanghai, China) following our previous procedure[4]. Total RNA was extracted from fresh frozen tissues using the Trizol reagent (Thermo Fisher Scientific, cat#15596018) and purified by removing rRNAs with NEBNext^®^ rRNA Depletion Kit (New England Biolabs Inc., Massachusetts, USA, cat#E6310X) following the manufacturer's instructions. All RNA analytes were assessed for fragment size, integrity, purity and concentration.

Subsequently, RNA libraries were constructed by using NEBNext^®^ Ultra^TM^ Ⅱ Directional RNA Library Prep Kit (New England Biolabs, Inc., Massachusetts, USA, cat#E6310) according to the manufacturer’s instructions. Libraries were controlled for quality and quantified using the Bioanalyzer 2100 system (Agilent Technologies, Inc., USA). Library sequencing was performed on an illumina Hiseq 4000 sequencer with 150 bp paired end mode. Raw reads were quality controlled by Q30. After removing sequencing adaptors, short reads (length < 35 bp) and low-quality reads with software of Cutadapt (v1.18) and Trimmomatic (v0.35), high-quality clean reads were obtained. Then, by using HISAT2 software (v2.1.0), clean reads of human data were aligned to the human genome (assembly GRCh38), while clean reads of mouse data were aligned to the mouse genome (assembly GRCh38). For the raw count data of genes and gene isoforms, R package edgeR (v3.26.8)[6] was used to filter lowly expressed items and to execute normalization to get reads per kilobase per million (RPKM) expression values. Differentially expressed mRNAs were identified by the cuts of *P* < 0.05, FDR q < 0.05 and fold change (FC) > 1.5 or FC < -1.5.

**Untargeted metabolomic profiling**

Samples from the control and FBL deficient ESCC cells were measured using the Pegasus High-Throughput Gas Chromatography with Time-of-Flight Mass Spectrometer system as we previously described[1]. While ESCC cells were examined using the Ultra High Performance Liquid Chromatography (1290 Infinity LC, Agilent Technologies) coupled to a tandem Mass Spectrometer system (TripleTOF 6600, AB Sciex) (UHPLC-MS/MS) by APT Biotechnology Company (Shanghai, China).

**Sample preparation**

Briefly, 200 μL cell supernatant was collected and thoroughly mixed with 800 μL metabolite extraction solution (methanol/acetonitrile, 1:1, vol/vol), followed by centrifugation at a speed of 14,000 × g at 4 ℃ for 20 minutes. Next, the acquired supernatant was dried in vacuum centrifuge. Finally, the residual was re-dissolved in 100 μL solvent (acetonitrile/water, 1:1, vol/vol), centrifuged at a speed of 14,000 × g at 4℃ for 20 minutes, and then injected for UHPLC-MS/MS analysis.

**UHPLC-MS/MS**

Samples were separated in a hydrophilic interaction chromatography (HILIC) column (2.1 mm × 100 mm ACQUIY UPLC BEH Amide 1.7 µm; Waters, Ireland) installed in the ultra-high performance liquid chromatography system. For each sample, 2 μL volume was injected into the column at 25℃ and at a flow rate of 0.5mL/min. The mobile phase, consisting of buffer A (25 mM ammonium acetate and 25 mM ammonia hydroxide dissolved in ultrapure water) and buffer B (acetonitrile), was employed in both ESI positive and negative modes. The gradient elution procedure was set as follows: 95% buffer B for 0.5 min, linearly reduced to 65% buffer B in 6.5 min, reduced to 40% buffer B in 1 min and kept for 1 min, and then increased to 95% buffer B in 0.1 min, with a 3 min re-equilibration period employed.

The ESI source conditions were set as follows: Ion source gas 1 as 60, Ion source gas 2 as 60, curtain gas as 30, source temperature at 600 ℃, and ionspray voltage floating at ± 5,500 V. In MS only acquisition, the instrument was set to acquire over the m/z range from 60 Da to 1,000 Da, and the accumulation time for TOF MS scan was set at 0.20 s/spectra. In auto MS/MS acquisition, the instrument was set to acquire over the m/z range from 25 Da to 1,000 Da, and the accumulation time for product ion scan was set at 0.05 s/spectra. The product ion scan was obtained using information dependent acquisition with high sensitivity mode selected. The parameters were set as follows: the collision energy at 35 V with ± 15 eV, de-clustering potential as 60 V (+) and −60 V (−), exclude isotopes within 4 Da, and candidate ions to monitor per cycle at 10.

**Untargeted metabolomic data analysis**

The raw data were converted to mzXML files using ProteoWizard software, and then imported into freely available XCMS software to pick peaks. During the calculation, parameters were set as follows: centWave m/z = 10 ppm, peakwidth = c (10, 60), prefilter = c (10, 100). For peak grouping, bw = 5, mzwid = 0.025, minfrac = 0.5. The R package, CAMERA (Collection of Algorithms of MEtabolite pRofile Annotation), was applied for annotating isotopes and adducts. In the extracted ion features, only the variables having more than 50% of nonzero measurement values in at least one group were kept. Metabolites were identified by comparing the accuracy m/z value (<10 ppm) and MS/MS spectra of peaks with an in-house database established with available authentic chemical standards. Heatmaps of metabolites between two groups were generated using the R package ComplexHeatmap [7].

**Differential abundance scores of metabolic pathways**

First, all measured metabolites were mapped onto KEGG pathways. After determining which metabolites were significantly increased/decreased in abundance, differential abundance (DA) scores of metabolic pathways were calculated using an algorithm as depicted previously [8]. DA captures the tendency for a pathway to have increased/decreased levels of metabolites, compared to a control group. DA score is defined as DA = (No. of metabolites increased － no. of metabolites decreased) / No. of measured metabolites in pathway.

**Download and analysis of the public GEO microarray dataset**

An GEO dataset, GSE23400, was downloaded from the NCBI Gene Expression Omnibus (http://www.ncbi.nlm.nih.gov/geo/). This dataset was normalized using the R function rma in the Bioconductor package affy and then log_2_-transformed. The *FBL* expression in ESCA was explored via GEPIA2 database (http://gepia2.cancer-pku.cn/).

**Statistical analysis**

Signiﬁcant differences between two groups were computed using Wilcoxon’s rank-sum test for data with skewed distribution or Student’s t test for data with normal distribution. Survival analysis was carried out by means of Kaplan-Meier method followed with log-rank test. Statistical analysis was conducted with R software (R version 3.6.1, http://www.r-project.org). All statistical tests were 2-tailed, and p values less than 0.05 were considered signiﬁcant.

**Figure S1. Related to Figure 1.**

**(A)** The protein levels of FBL between matched ESCC tumor tissues and normal adjacent tissues in validation cohort. **(B)** Disease-free survival curves of ESCC patients of cohort 1 (n = 90) stratified by low and high FBL expressions from IHC staining. Survival p values were computed by using log-rank test. **p* < 0.05, ***p* < 0.01, ****p* < 0.001, paired Wilcoxon’s rank-sum test.

**Figure S2. Related to Figure 2**

**(A)** *FBL* deletion in ESCC KYSE150 and Eca109 cells via the CRISPR-Cas9 system. **(B)** Impact of *FBL* deletion on ESCC cell growth. **(C)** Flow cytometric analysis revealing G1 phase arrest of ESCC cells with *FBL* deletion. **(D)** Colony formation in soft agar demonstrating impaired growth in *FBL*-deficient cells. **(E)** Ectopic FBL expression in ESCC cells via the CRISPR-SAM system. **(F)** The impact of FBL overexpression on ESCC cell growth. **(G)** Colony formation in soft agar revealing expedited growth of ESCC cells with FBL overexpression. Error bars represent mean ± SEM. **p* < 0.05, ***p* < 0.01, ****p* < 0.001, Student’s t-test.

**Figure S3. Related to Figure 2**

**(A)** *FBL* ablation curtailed the growth of KYSE150 xenograft tumors. **(B, C)** Images and weights of KYSE150 xenograft tumors with or without *FBL* deletion. **(D)** *FBL* ablation curtailed the growth of Eca109 xenograft tumors. **(E, F)** Images and weights of Eca109 xenograft tumors with or without *FBL* deletion. Error bars represent mean ± SEM. **p* < 0.05, ***p* < 0.01, ****p* < 0.001, Student’s t-test.

**Figure S4. Related to Figure 2.**

**(A)** Cell growth of WT KYSE150 cells when mixed with control KYSE150 cells (GFP) or *FBL*-deficient KYSE150 cells (Td-tomato), respectively. **(B)** Cell counting of WT KYSE150 cells mixed with control KYSE150 cells (GFP) or *FBL*-deficient KYSE150 cells (Td-tomato). Error bars represent mean ± SEM. **p* < 0.05, ***p* < 0.01, ****p* < 0.001, Student’s t-test.

**Figure S5. Related to Figure 3.**

**(A)** Principal component analysis (PCA) score plots of metabolomic data of control and *FBL*-deficient KYSE150 cells. **(B)** Heatmap showing all significantly altered metabolites in *FBL*-deficient KYSE150 cells as relative to control cells. These metabolites were classified by chemical taxonomy. **(C)** Comparison of the essential enzymes participated in the downstream metabolism of NAD between ESCC tissues and adjacent normal tissues by using RNA sequencing data of discovery cohort. **p* < 0.05; ns, no significance; paired Wilcoxon’s rank-sum test.

**Figure S6. Related to Figure 4.**

**(A)** The protein levels of SIRT1 between paired ESCC tumor tissues and adjacent normal tissues in validation cohort. **(B)** Absence of *FBL* significantly accelerated the protein degradation of SIRT1 under cycloheximide treatment in Eca109 cells. **(C)** Western blot assay exhibiting SIRT1expression following knockdown of its putative E3 ubiquitin ligases in *FBL*-deficient cells**.** **(D)** CHX chase assay demonstrating the degradation rate SIRT1 in control *FBL*-deficient ESCC cells or *FBL*-deficient ESCC cells with *smurf2* deletion. **(E**) OP-Puro incorporation assay revealing global protein translation activity in control and *FBL*-deficient ESCC cells. ****p* < 0.001, paired Wilcoxon’s rank-sum test.

**Figure S7. Related to Figure 6.**

**(A-B)** Cox proportional hazards regression analyses showing the independent prognostic value of SIRT1 and FBL for OS prediction after adjustment for age, sex, and TNM stage. Cohort 2 was enrolled for analyses.

**Figure S8. Related to Figure 7.**

**(A)** Compromised efficacy of CGX-635 in *FBL*-deleted cells compared with control cells. **(B and C)** Color-gradient plots of the dose response matrix (B) and HSA synergy scores (C) quantitatively showing the synergistic effect between CGX-635 and ABT-263. The results were derived from a highest single agent synergy (HSA) model. **(D–F)** Influence of CGX-635 and ABT-263, alone or in combination, on AKR xenograft tumor growth in an immunocompetent C57BL/6 mouse model. Tumor growth curves, statistical analysis of tumor weights, and tumor images were presented in D, E, and F, respectively. ABT-263 was administered by intragastric gavage at a dose of 25 mg/kg every 3 days, while CGX-635 was given by intraperitoneal injection at a dose of 0.5 mg/kg/day. **(G and H)** Impact of CGX-635 and ABT-263, alone or in combination, on mouse body weights. Bothe an immunodeficient mouse model constructed with KYSE150 cells and an immunocompetent mouse model established with ARK cells were used for analysis. **(I and J)** Influence of CGX-635 and ABT-263, alone or in combination, on the histopathological architecture of major organs. Both an immunodeficient mouse model constructed with KYSE150 cells and an immunocompetent mouse model established with ARK cells were used for analysis. Error bars represent mean ± SEM. *p < 0.05, **p < 0.01, ***p < 0.001, Student’s t-test.

**REFERENCES**

1.	Jin, X., et al., *A multi-omics study delineates new molecular features and therapeutic targets for esophageal squamous cell carcinoma.* Clin Transl Med, 2021. **11**(9): p. e538.

2.	Liu, L., et al., *New Metabolic Alterations and A Predictive Marker Pipecolic Acid in Sera for Esophageal Squamous Cell Carcinoma.* Genomics Proteomics Bioinformatics, 2022. **20**(4): p. 670-687.

3.	Liu, D., et al., *Active post-transcriptional regulation and ACLY-mediated acetyl-CoA synthesis as a pivotal target of Shuang-Huang-Sheng-Bai formula for lung adenocarcinoma treatment.* Phytomedicine, 2023. **113**: p. 154732.

4.	Chen, W.L., et al., *GLUT5-mediated fructose utilization drives lung cancer growth by stimulating fatty acid synthesis and AMPK/mTORC1 signaling.* JCI Insight, 2020. **5**(3).

5.	Liu, D., et al., *Oleanolic acid blocks the purine salvage pathway for cancer therapy by inactivating SOD1 and stimulating lysosomal proteolysis.* Mol Ther Oncolytics, 2021. **23**: p. 107-123.

6.	Robinson, M.D., D.J. McCarthy, and G.K. Smyth, *edgeR: a Bioconductor package for differential expression analysis of digital gene expression data.* Bioinformatics, 2010. **26**(1): p. 139-40.

7.	Gu, Z., R. Eils, and M. Schlesner, *Complex heatmaps reveal patterns and correlations in multidimensional genomic data.* Bioinformatics, 2016. **32**(18): p. 2847-9.

8.	Hakimi, A.A., et al., *An Integrated Metabolic Atlas of Clear Cell Renal Cell Carcinoma.* Cancer Cell, 2016. **29**(1): p. 104-116.
